# Beta-blocker efficacy across different cardiovascular indications: an umbrella review and meta-analytic assessment

**DOI:** 10.1186/s12916-020-01564-3

**Published:** 2020-05-05

**Authors:** Oliver J. Ziff, Monica Samra, James P. Howard, Daniel I. Bromage, Frank Ruschitzka, Darrel P. Francis, Dipak Kotecha

**Affiliations:** 1 University of Birmingham Institute of Cardiovascular Sciences, Medical School, Birmingham, B15 2TT UK; 2 University College London, London, WC1E 6BT UK; 3 Imperial College London, London, SW7 2AZ UK; 4 Kings College London, London, WC2R 2LS UK; 5 University Hospital Zurich, 8091 Zürich, Switzerland; 6 Monash Centre of Cardiovascular Research & Education in Therapeutics, Monash University, Melbourne, Victoria 3004 Australia; 7 University Hospitals Birmingham NHS Foundation Trust, Queen Elizabeth Hospital, Institute of Translational Medicine, Birmingham, B15 2GW UK

**Keywords:** Systematic review, Coronary artery disease, Heart failure, Hypertension, Perioperative, Meta-analysis, Mortality, Myocardial infarction, Stroke

## Abstract

**Background:**

Beta-blockers are widely used for many cardiovascular conditions; however, their efficacy in contemporary clinical practice remains uncertain.

**Methods:**

We performed a prospectively designed, umbrella review of meta-analyses of randomised controlled trials (RCTs) investigating the evidence of beta-blockers in the contemporary management of coronary artery disease (CAD), heart failure (HF), patients undergoing surgery or hypertension (registration: PROSPERO CRD42016038375). We searched MEDLINE, EMBASE and the Cochrane Library from inception until December 2018. Outcomes were analysed as beta-blockers versus control for all-cause mortality, myocardial infarction (MI), incident HF or stroke. Two independent investigators abstracted the data, assessed the quality of the evidence and rated the certainty of evidence.

**Results:**

We identified 98 meta-analyses, including 284 unique RCTs and 1,617,523 patient-years of follow-up. In CAD, 12 meta-analyses (93 RCTs, 103,481 patients) showed that beta-blockers reduced mortality in analyses before routine reperfusion, but there was a lack of benefit in contemporary studies where ≥ 50% of patients received thrombolytics or intervention. Beta-blockers reduced incident MI at the expense of increased HF. In HF with reduced ejection fraction, 34 meta-analyses (66 RCTs, 35,383 patients) demonstrated a reduction in mortality and HF hospitalisation with beta-blockers in sinus rhythm, but not in atrial fibrillation. In patients undergoing surgery, 23 meta-analyses (89 RCTs, 19,211 patients) showed no effect of beta-blockers on mortality for cardiac surgery, but increased mortality in non-cardiac surgery. In non-cardiac surgery, beta-blockers reduced MI after surgery but increased the risk of stroke. In hypertension, 27 meta-analyses (36 RCTs, 260,549 patients) identified no benefit versus placebo, but beta-blockers were inferior to other agents for preventing mortality and stroke.

**Conclusions:**

Beta-blockers substantially reduce mortality in HF patients in sinus rhythm, but for other conditions, clinicians need to weigh up both benefit and potential risk.

## Introduction

Beta-blockers are an established part of the routine management for many cardiovascular conditions and are widely used by physicians across the spectrum of healthcare. They act via multiple pathways, limiting the effects of catecholamine excess, affecting inotropy and chronotropy, providing anti-arrhythmic and anti-ischaemic effects and inhibiting renin release. However, this diversity of action carries the possibility of varying net effects on individual outcomes, depending on patient characteristics and disease substrate. Beta-blockers are well tolerated even in patients with advanced conditions such as heart failure with reduced ejection fraction (HFrEF), as confirmed in double-blind randomised controlled trials (RCTs) [1]. Although side effects such as dizziness, lethargy and cold extremities are commonly reported in clinical practice, similar rates are seen with placebo [2]. Beta-blockers can cause bradycardia, atrioventricular block and symptomatic hypotension, especially in patients with sinus and/or atrioventricular node dysfunction [3], and are contraindicated in severe asthma because of the risk of life-threatening bronchospasm [4].

The balance of risk versus benefit remains unclear in many cardiovascular conditions. For example, in acute coronary syndromes (ACS) and patients with coronary artery disease (CAD), many RCTs predate reperfusion strategies and contemporary medical treatment [5, 6]. There have also been questions about the efficacy of beta-blockers in HFrEF patients with concomitant atrial fibrillation [7] and safety concerns for patients undergoing non-cardiac surgery and in hypertension [8–10]. These uncertainties have contributed to suboptimal uptake in patient groups where beta-blockers are known to reduce mortality and morbidity. Indeed, patients at greatest risk of death seem least likely to receive evidence-based therapy [11, 12]. The aim of our study was to review all available evidence for beta-blocker therapy across these different cardiovascular indications, providing clear assistance for clinicians and to inform future guidelines. For each condition, we used an evidence-based hierarchy that first identified the availability of individual patient data (IPD) meta-analyses, and if not, whether there were aggregate data meta-analyses with unbiased data to better understand the balance of efficacy and safety of beta-blockers.

## Methods

Due to the comprehensive assessment, the main text focuses on the most robust findings with clinical impact; all additional data are available in the Online Data Supplement. The project was prospectively registered with the PROSPERO database of systematic reviews (CRD42016038375, [13]) and conducted according to the Preferred Reporting Items for Systematic reviews and Meta-Analyses (PRISMA) guidelines [14]. A systematic review of MEDLINE, EMBASE, the Cochrane Library and other sources was performed (1960 to December 2018). Additional details on methodology are presented in the Supplemental Methods.

### Study selection

We included meta-analyses of RCTs that looked at the clinical effects of beta-blockers on adults in four cardiovascular indications: CAD, heart failure, perioperative risk reduction and hypertension. Definitions of cardiovascular conditions and outcomes used by each individual meta-analysis were accepted and are reported in Supplemental Table 1. Administration of beta-blockers was via any route versus control (placebo or no treatment). In the hypertension population, we also included meta-analyses comparing beta-blockers with another active drug. Supplemental Tables 1 and 2 provide details and references for all included and excluded meta-analyses, respectively.

### Data extraction

The predefined primary outcome was all-cause mortality. Secondary outcomes were cardiovascular mortality, incident myocardial infarction (MI), heart failure and stroke, as relevant to the cardiovascular indication.

### Quality assessment

We employed an evidence-based hierarchy to determine the quality of data. The first stage was the type of meta-analysis, with IPD meta-analyses ranking first, and then aggregate tabular data meta-analyses (herein simply referred to as meta-analyses). The second stage involved careful exploration of study quality and potential bias using AMSTAR (A Measurement Tool to Assess Multiple Systematic Reviews) and ROBIS (Risk of Bias in Systematic Reviews, Supplemental Table 3). The certainty of evidence was evaluated using the GRADE (Grading of Recommendations Assessment, Development and Evaluation) approach and was classified as high, moderate, low or very low (Supplemental Table 4 and using the GRADEproGDT software at gdt.gradepro.org/app/) [15].

### Data synthesis and analysis

The outcome data from each meta-analysis was summarised as a risk ratio (RR) using an intention-to-treat approach and graphed in a forest plot. RR and associated 95% confidence intervals (CI) were calculated using the published outcome data. These effect estimates were not pooled, as many of the meta-analyses within the same indication included the same trials; instead, we display the range of confidence intervals and highlight the highest quality/lowest bias estimates of beta-blockers versus control. Heterogeneity across trials is displayed as the *I*^2^ statistic. A two-tailed *p* value of 0.05 was considered statistically significant. Analyses were performed on Stata version 14.1 (StataCorp LP, Texas).

## Results

We identified 98 meta-analyses suitable for quantitative synthesis. These included 284 unique RCTs, with 418,624 unique patients on beta-blocker therapy or control, and 1,617,523 patient-years of follow-up (Supplemental Figure 1). The risk of bias was variable in the meta-analyses, but generally low (Supplemental Table 3 and Supplemental Figure 2). There were notable exceptions, including potential bias in data collection and study selection in heart failure, and moderate overall risk of bias in hypertension studies. Quality and risk of bias corresponded to the robustness of the study design, with IPD and prospectively registered meta-analyses demonstrating the highest quality and lowest risk of bias.

### Coronary artery disease

**Fig. 1:**
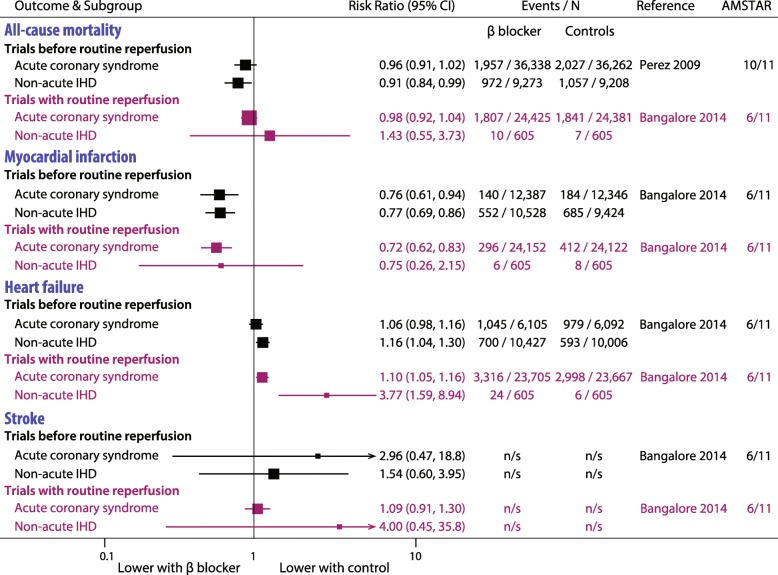
Representative coronary artery disease meta-analyses.Most up-to-date and highest quality meta-analyses for coronary artery disease. No individual patient data meta-analyses were available. Trials with routine reperfusion had ≥ 50% of patients receiving thrombolytics or intervention. See Supplemental Figures 3 for full details. IHD, ischaemic heart disease

There were no IPD studies and 12 other meta-analyses meeting inclusion criteria, comprising 93 individual RCTs, 103,481 participants, mean follow-up of 7.8 months (range: in-hospital to 6 years) and a total of 55,536 patient-years of follow-up. The RCTs were predominantly historical, being performed before the routine use of reperfusion strategies; only 8 trials included patients where ≥ 50% of patients received thrombolytics or intervention. A summary of the most contemporary data is presented in Fig. 1 and detailed analysis in Supplemental Figure 3.

#### All-cause mortality

In studies before routine reperfusion, beta-blockers reduced mortality compared to control in acute meta-analyses (within 48 h of MI, RR range 0.83 to 0.98) and non-acute meta-analyses (after 48 h, RR range 0.38 to 0.96). Where sub-group analyses were performed to assess trials with routine reperfusion (where ≥ 50% of patients received thrombolytics or intervention), beta-blockers did not reduce mortality either in the acute setting (12 RCTs, 48,806 participants; RR 0.98, 95% CI 0.92–1.04) or in non-acute MI (2 RCTs, 1210 participants; RR 1.43, 95% CI 0.55–3.73).

#### Incident MI

In trials before routine reperfusion, beta-blockers reduced MI in both the acute setting (48 RCTs with 24,773 participants; RR 0.76, 95% CI 0.61–0.94) and non-acute setting (18 RCTs with 20,637; RR 0.77, 95% CI 0.69–0.86). Beta-blockers reduced incident MI in studies with routine reperfusion only in the acute setting (12 RCTs with 48,274 participants, RR 0.72, 95% CI 0.62–0.83; non-acute setting 2 RCTs with 1210 participants, RR 0.75, 0.26–2.15).

#### Heart failure

In the acute setting, incident heart failure tended to be more common with beta-blockers than control, but this was only significant in trials with routine reperfusion (9 RCTs with 47,272 participants; RR 1.10, 95% CI 1.05–1.16). Beta-blockers increased incident heart failure in the non-acute setting in trials both before routine reperfusion (17 RCTs with 20,433 participants; RR 1.16, 95% CI 1.04–1.30) and with routine reperfusion (2 RCTs with 1210 participants; RR 3.77, 95% CI 1.59–8.94).

#### Stroke

In both the acute and non-acute setting, beta-blockers had no effect on incident stroke either before routine reperfusion (acute RR 2.96, 95% CI 0.47–18.81; non-acute RR 1.54, 95% CI 0.60–3.95), or with routine reperfusion (acute RR 1.09, 95% CI 0.91–1.30; non-acute RR 4.00, 95% CI 0.45–35.79).

### Heart failure

**Fig. 2:**
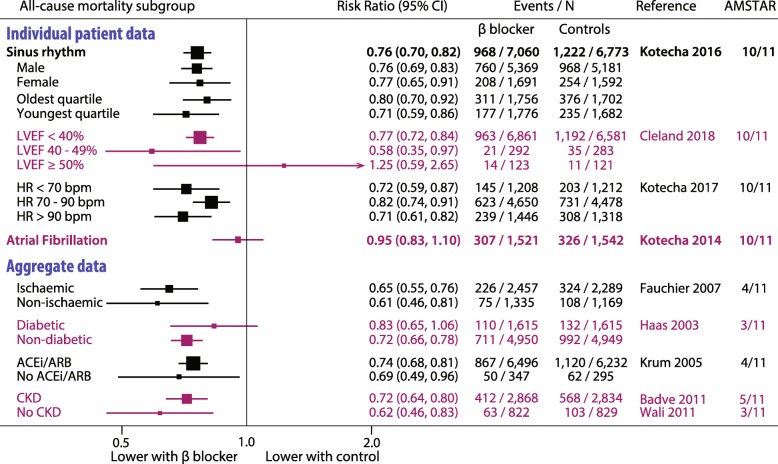
Representative heart failure meta-analyses for all-cause mortality.Most up-to-date and highest quality meta-analyses for heart failure. Results for other outcomes were similar to all-cause mortality. See Supplemental Figure 4 for full details. ACEi, angiotensin-converting enzyme inhibitors; ARB, angiotensin receptor blockers; bpm, beats/min; CKD, chronic kidney disease; HR, heart rate; LVEF, left ventricular ejection fraction

Four IPD studies and 31 other meta-analyses met inclusion criteria, comprising 66 individual RCTs, 35,383 participants, mean follow-up of 12.3 months (range 3 to 32 months) and a total of 534,461 patient-years of follow-up. The RCTs were predominantly in patients with HFrEF; only 3 trials were designed to include patients with left ventricular ejection fraction (LVEF) > 40%. A summary of the highest quality data is presented in Fig. 2 and detailed analysis in Supplemental Figure 4.

#### All-cause mortality

IPD meta-analysis of 11 double-blind, placebo-controlled RCTs demonstrated that beta-blockers significantly reduced mortality in patients with sinus rhythm (13,833 patients; RR 0.76, 95% CI 0.70–0.82), regardless of age, gender or achieved heart rate. However, there was no reduction in mortality in the subgroups with atrial fibrillation (3064 patients; RR 0.95, 0.83–1.10; *p*_interaction_ 0.002 for rhythm on baseline electrocardiogram) [7], or the small subgroup with LVEF ≥ 50% [16]. In non-IPD meta-analyses, beta-blockers were associated with reduced mortality compared to control, but findings were non-significant in subgroups with atrial fibrillation, LVEF > 40% and black patients, and inconsistent in patients with diabetes.

#### Cardiovascular mortality, heart failure hospitalisation and stroke

In both IPD and other meta-analyses, patients in (or predominantly in) HFrEF and sinus rhythm had substantial reduction in cardiovascular mortality and heart failure hospitalisation with beta-blockers compared to control. Beta-blockers had no effect on non-fatal stroke compared with placebo (16,644 patients) in patients in HFrEF, irrespective of if they were in sinus rhythm (HR 1.02, 95% CI 0.78–1.32) or atrial fibrillation (HR 1.04, 95% CI 0.66–1.63).

### Perioperative risk reduction

**Fig. 3:**
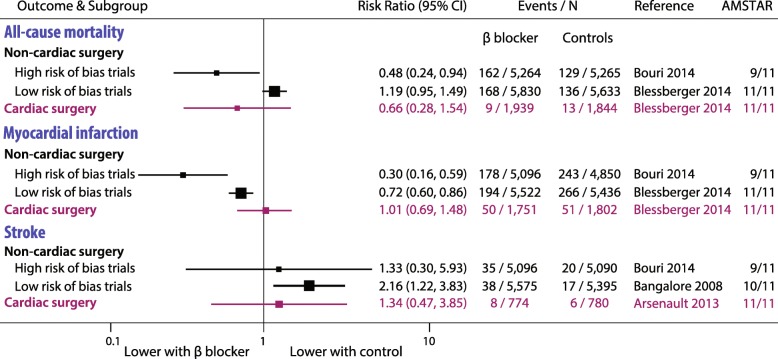
Representative perioperative meta-analyses.Highest quality meta-analyses of perioperative risk reduction using beta-blockers. No individual patient data meta-analyses were available. See Supplemental Figure 5 for full details. The Dutch Echocardiographic Cardiac Risk Evaluation Applying Stress Echocardiography (DECREASE) trials II–VI were considered high risk of bias

There were no IPD studies. Twenty-three other meta-analyses with 89 individual RCTs were included, with 19,211 participants followed-up for a mean of 1 month (range 24 h to 2 years; 1925 patient-years of follow-up). A summary of representative data is presented in Fig. 3 and detailed analysis in Supplemental Figure 5.

#### All-cause mortality

In non-cardiac surgery, we saw a clear distinction in treatment effect according to the risk of bias. In small meta-analyses with high risk (that included the DECREASE studies), there were reductions in mortality with beta-blockers. Conversely, all large meta-analyses with low risk of bias in non-cardiac surgery suggested that beta-blockers increased mortality compared to control, ranging from RR 1.03 to 1.31. Meta-analyses in patients undergoing cardiac surgery showed no significant effect of beta-blocker therapy.

#### Incident MI and stroke

Beta-blockers were consistently associated with a reduced risk of incident MI after non-cardiac surgery (point estimate RR range 0.08 to 0.92), but an increased risk of stroke (RR range 1.33 to 7.72). None of the meta-analyses in cardiac surgery identified any significant difference with beta-blockers or control for either incident MI or stroke.

### Hypertension

**Fig. 4:**
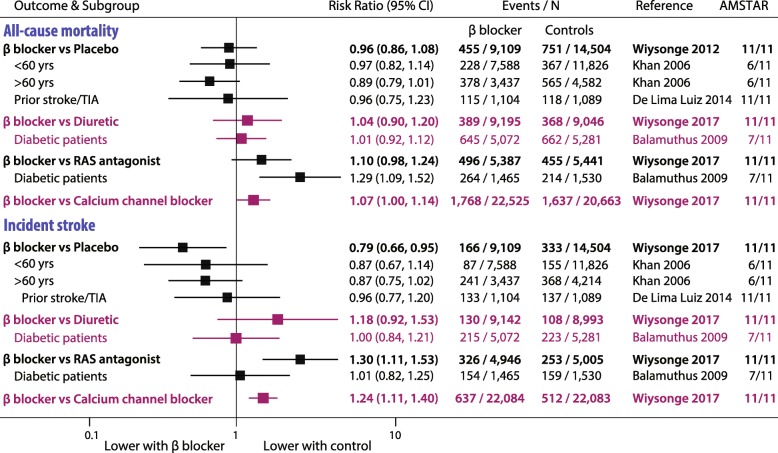
Representative hypertension meta-analyses.Highest quality meta-analyses of hypertension using beta-blockers. No individual patient data meta-analyses were available. See Supplemental Figure 6 for full details. RAS, renin-angiotensin system; TIA, transient ischaemic attack

There were no IPD studies and 28 other meta-analyses, with 36 individual RCTs and 260,549 participants followed up for a mean of 3.7 years (range 1 to 10 years; 1,025,601 patient-years of follow-up). Summary findings are displayed in Fig. 4 and full analysis in Supplemental Figure 6.

#### All-cause mortality

Mortality was not significantly different in any of 18 meta-analyses comparing beta-blockers with either placebo or diuretics. Compared to renin-angiotensin system (RAS) antagonists, all 5 meta-analyses demonstrated a trend to increased mortality with beta-blockers, ranging from RR 1.08 to 1.29, with diabetic patients showing a significant increase. Compared to CCB, all 3 meta-analyses demonstrated significantly increased mortality with beta-blockers (RR range 1.07 to 1.11).

#### Incident MI and stroke

Across meta-analyses, there were no consistent differences in incident MI comparing beta-blockers with either placebo or other therapy. Incident stroke rates were significantly increased with beta-blockers versus either RAS antagonists or CCB (representative RR 1.30 and 1.24, respectively), although not in diabetic patients.

#### Sensitivity analysis

There were no consistent differences between atenolol and non-atenolol beta-blockers for the primary or secondary outcomes (Supplemental Figure 7).

## Discussion

**Fig. 5:**
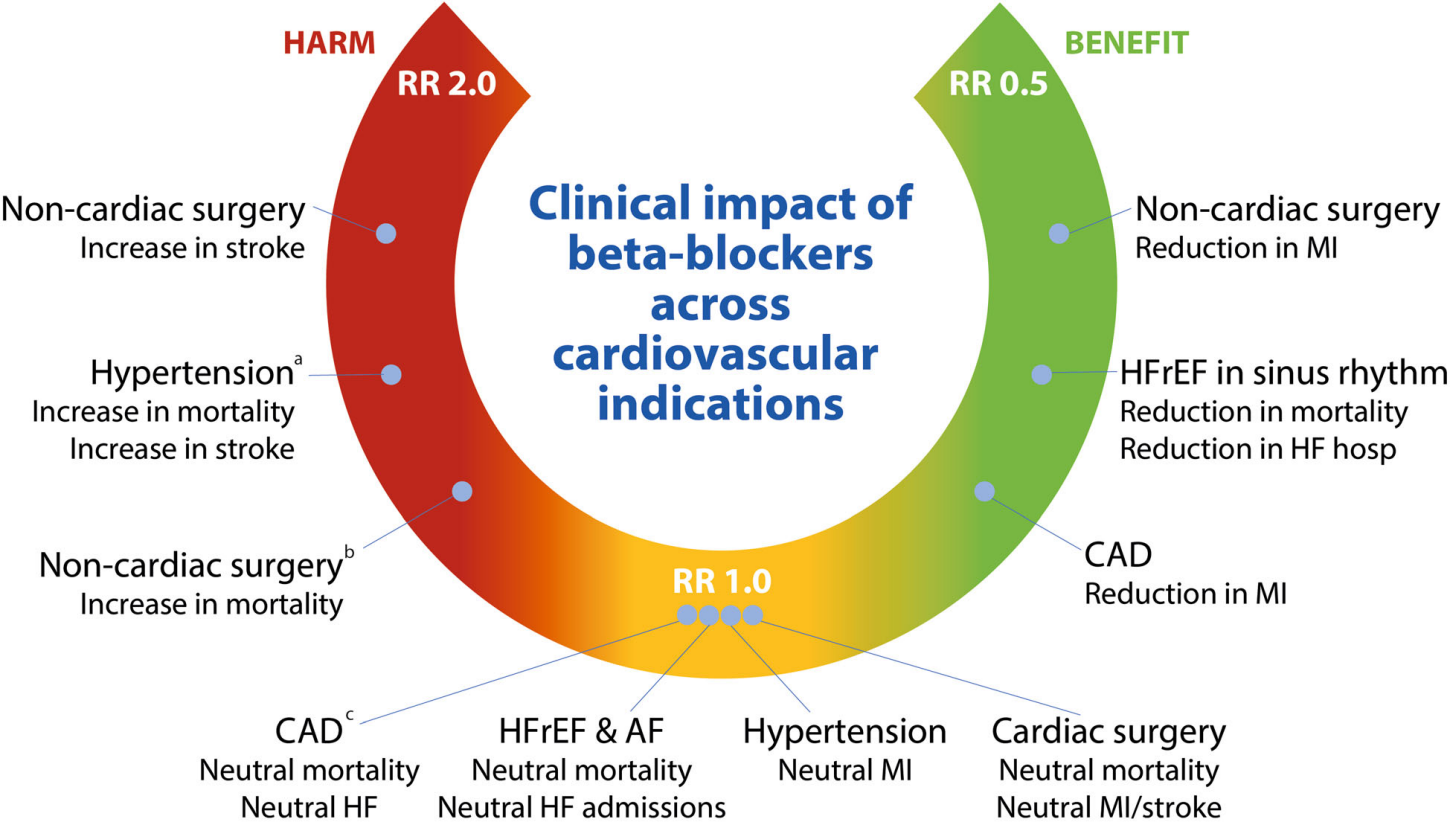
Overview of the evidence base for beta-blockers versus control in cardiovascular health.**a** Compared to other medications. **b** In trials with a low risk of bias. **c** In contemporary trials with majority undergoing reperfusion. AF, atrial fibrillation; CAD, coronary artery disease; HF, heart failure; HFrEF, heart failure with reduced ejection fraction; MI, myocardial infarction; RR, risk ratio

This umbrella review across cardiovascular indications used meta-analytic data to clarify the evidence basis for beta-blockers in contemporary clinical practice (Table 1, Fig. 5). In patients with coronary disease, there is a trade-off between benefit and risk. Beta-blockers reduced the risk of incident MI at the expense of higher rates of incident HF, with no effect on mortality. In patients with HFrEF in sinus rhythm, beta-blockers reduced morbidity and mortality, but they had no effect in HFrEF with concomitant atrial fibrillation or those with preserved LVEF. In perioperative patients, although beta-blockers reduced the risk of incident MI in those undergoing non-cardiac surgery, this was at the expense of increased mortality and stroke. Beta-blockers had no effect on any of the outcomes assessed in patients undergoing cardiac surgery. In hypertension, although beta-blockers had no significant effect compared to placebo or diuretics, they were inferior to RAS antagonists and CCB. These findings highlight the importance of an individualised assessment of indication, comorbidity and understanding of the goal of therapy before routine commencement of beta-blockers.

**Table 1:**
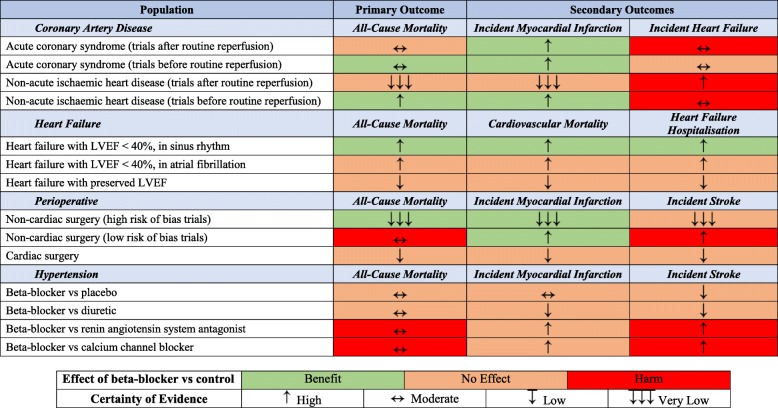
Evidence map of availability and appraisal of certainty of evidence for beta-blockers across cardiovascular indications

Certainty of evidence was assessed using the GRADE guidelines. See Supplemental Table 4 for full details. LVEF, left ventricular ejection fraction

### Coronary artery disease

Although beta-blockers were associated with reduction in mortality in trials before routine reperfusion, the reduction in MI was offset by an increased risk of heart failure. Although MI was a relatively rare event (absolute event rate ~ 0.01%) and heart failure was more common (absolute event rate ~ 13%), the number needed to treat (NNT) to prevent an MI was 118 compared with the number needed to harm (NNH) of 127 to cause an incident heart failure event. Additionally, before routine reperfusion, the event rate for mortality was 7.8% in control versus 6.7% with beta-blockers (NNT 89) [6]. There were less tangible benefits in trials with routine reperfusion where the majority of patients received reperfusion with either thrombolytics or coronary intervention. In the International Study of Infarct Survival (ISIS-1) trial in 1986, atenolol significantly reduced vascular death, but only 5% of patients were on antiplatelet agents [17]. In contrast, in the large Clopidogrel and Metoprolol in Myocardial Infarction Trial (COMMIT) published in 2005, metoprolol failed to reduce mortality where all patients were on aspirin, 50% on dual antiplatelet agents and 66% received thrombolysis [18]. The combination of antiplatelet agents with prompt reperfusion therapy in the modern era may restrict the extent of myocardial damage in patients with MI and hence limit the substrate which can benefit from sympathetic inhibition and reduced myocardial oxygen demand.

The balance of benefit versus risk is particularly relevant in acute MI where negative inotropy can lead to cardiogenic shock. This may be more pertinent in larger ST-elevation MI, where early high-dose beta-blockade was associated with increased mortality [18]. In lower-risk unstable angina patients, beta-blockers reduce the risk of progression to acute MI [19]. Treatment duration may be an important factor to achieve longer-term benefit, but no RCTs directly investigated this issue. The prognostic effects of beta-blockers in chronic stable ischaemic heart disease and stable angina without prior MI remain unanswered. These issues all have important relevance for future guidelines, particularly whether historical data should be used to support contemporary recommendations.

### Heart failure

Beta-blockers have a class I, level of evidence A recommendation in chronic HFrEF, and yet uptake in clinical practice remains suboptimal in certain groups [20, 21]. Subgroup analyses have demonstrated that reduction of mortality and hospitalisation with beta-blockers is consistent in sinus rhythm, regardless of age or gender, baseline heart rate or the degree of reduction in ejection fraction [1, 16, 22]. However, IPD meta-analysis revealed no signal for benefit in the subgroup of patients with atrial fibrillation at baseline [7]. The reasons for this distinction remain unclear, but may relate to differences in the association of heart rate and prognosis compared with sinus rhythm [22], or consequences such as myocardial fibrosis that may influence therapeutic efficacy [23]. Heart failure with intermediate and preserved LVEF accounts for over half of patients, and yet available data is extremely limited [16]. Further RCTs are clearly warranted to address this evidence gap, as well as the interaction with renal dysfunction which is common in patients with HF [24].

### Perioperative risk reduction

Beta-blockers are commonly considered to reduce the risk of cardiovascular events in patients undergoing surgery, but the controversy over integrity of data in the DECREASE studies has led to questions about their true value [25]. In meta-analyses with low risk of bias, we saw an increase in mortality following non-cardiac surgery compared with control and consistent increases in stroke risk. These adverse events offset any benefit from the reduced risk of MI and were consistent with the largest and highest quality individual RCT, the PeriOperative ISchemic Evaluation (POISE) trial [26]. However, the frequency of mortality and incident stroke (absolute event rates ~ 0.3% and ~ 0.6%, respectively) were rarer than myocardial infarction (~ 4%). Indeed, the NNT to prevent an MI was 72 compared with a NNH of 214 to cause a death and 352 to cause a stroke [27]. In cardiac surgery, where one might anticipate a prognostic benefit from beta-blockers, we found no appreciable reduction in events, including for incident MI.

### Hypertension

Beta-blockers are no longer considered as a preferred initial therapy for essential hypertension [9]. The changing approach and therapeutic options are further clarified in the most recent guidelines [10]. However, many patients still receive beta-blockers or continue therapy from historical prescriptions [28]. Our analysis confirms that beta-blockers are inferior to other agents (most notably CCB and RAS antagonists), particularly in preventing strokes. This may be partly explained by differences in achieved BP, including central systolic BP between different drug treatments, to which cerebrovascular events may be particularly sensitive. In addition, beta-blockers are somewhat less effective than RAS antagonists and CCB in preventing target organ damage.

Importantly, beta-blockers are a heterogeneous class. Whilst vasodilating beta-blockers, such as nebivolol, have demonstrated favourable effects on central BP and cardiovascular surrogates, RCTs with these newer beta-blockers in hypertensive patients are currently lacking. It is possible that there are important differences between beta-blockers in efficacy and safety; however, there are insufficient data to be certain as atenolol was utilised in ~ 75% of trials (our sensitivity analysis could only group together ‘non-atenolol’ studies). Although there remains a place for beta-blockers in resistant hypertension or those with cardiovascular comorbidities, our data would suggest that physicians should be more aggressive in switching to alternative classes of anti-hypertensive medication.

### Strengths and limitations

The strength of this analysis was the use of a systematic and methodical approach to assess all published meta-analyses of randomised trials across different cardiovascular diseases. By focusing on the highest quality data through a detailed risk of bias and quality assessment, we were able to come to conclusions across various cardiovascular conditions and subgroups. The trial populations were heterogeneous, but this provides results that are more likely to represent clinical practice. It would not have been feasible to assess all individual RCTs due to the vast number included in our analysis. Our analysis provides the most comprehensive and contemporary summary of the efficacy of beta-blockers across cardiovascular diseases, thereby aiding physicians to initiate, maintain or withdraw therapy in a patient group that frequently has multi-morbidity and more than one clinical indication.

As with all systematic reviews, we are limited by the individual RCTs and the methodology of the component meta-analyses. IPD has important advantages over aggregate data meta-analysis, including the ability to improve data quality and increase the precision of effect size. Unfortunately, the series of IPD analyses from the Beta-blockers in Heart Failure Collaborative Group [29] were the only IPD studies available. Nevertheless, at least for major outcomes, conventional meta-analysis can provide similar results and conclusions [30].

There are cardiovascular indications for beta-blockers where data are extremely limited. Beta-blockers are commonly used to control heart rate in patients with atrial fibrillation; however, the evidence base is extremely limited at present [31] and we identified no studies eligible for inclusion. Other cardiac populations where beta-blockers are regularly prescribed but where randomised trial evidence is limited include chemotherapy toxicity, cardiomyopathies and other arrhythmias (atrial and ventricular tachycardia).

There are also important and complex pharmacokinetic and pharmacodynamic differences between beta-blocker subtypes, including lipid solubility, intravenous versus oral administration routes, bioavailability and first-pass metabolism variations. However, with the exception of hypertension, we were unable to assess heterogeneity of treatment effects between different beta-blockers [32]. Finally, the trade-off between efficacy and adverse events will vary according to individual patient characteristics (cardiovascular condition, disease severity and course, comorbidities, and coexisting therapies) and beta-blocker effects (pharmacodynamics, receptor selectivity, dose and route of administration). All of these factors, and more, will impact on the clinical decision to prescribe beta-blocker therapy.

## Conclusions

Although beta-blockers are widely used in routine clinical practice, this analysis indicates that their overall clinical effect depends strongly on the clinical situation. In patients with heart failure and reduced ejection fraction who are in sinus rhythm, beta-blockers show clear benefit in terms of mortality reduction and lower rates of hospitalisation. However, beta-blockers show neutral effects for many other clinical situations in the modern era, and in some cases, harm.

## Supplementary information

**Additional file 1.**

## Data Availability

All data generated or analysed during this study are included in this published article [and its supplementary information files].
