# Supplementary material for: Beta-blocker efficacy across different cardiovascular indications: an umbrella review and meta-analytic assessment
Source: BMC Med. 2020 May 5;18:103. doi: 10.1186/s12916-020-01564-3 (PMC7199339; doi:10.1186/s12916-020-01564-3)
Supplement: Supplementary file 1 — Additional file 1. [file 12916_2020_1564_MOESM1_ESM.pdf]

SUPPLEMENTAL MATERIAL

Beta-blocker efficacy across different cardiovascular indications: A systematic and meta-analytic assessment.

BMC Medicine 2020; DOI : 10.1186/s12916-020-01564-3

Oliver J. Ziff, Monica Samra, James P. Howard, Daniel I. Bromage, Frank Ruschitzka, Darrel P. Francis and Dipak Kotecha*

SUPPLEMENTAL METHODS 2

SEARCH STRATEGY 2

DATA COLLECTION, ANALYSIS AND EXTRACTION OF META-ANALYSES 3

RISK OF BIAS AND QUALITY ASSESSMENT 3

ADDITIONAL STATISTICAL METHODS 3

SUPPLEMENTAL TABLES 4

SUPPLEMENTAL TABLE 1: DETAILS OF INCLUDED META-ANALYSES 4

SUPPLEMENTAL TABLE 2: DETAILS OF EXCLUDED META-ANALYSES 12

SUPPLEMENTAL TABLE 3: ROBIS RESULTS FOR EACH INDIVIDUAL SYSTEMATIC REVIEW AND META-ANALYSIS 16

SUPPLEMENTAL TABLE 4: GRADE SCALE FOR ASSESSMENT OF CERTAINTY OF EVIDENCE 20

SUPPLEMENTAL FIGURES 23

SUPPLEMENTAL FIGURE 1. PRISMA FLOW DIAGRAM 23

SUPPLEMENTAL FIGURE 2. ROBIS RESULTS FROM META-ANALYSES IN EACH CARDIOVASCULAR CONDITION 24

SUPPLEMENTAL FIGURE 3: CORONARY ARTERY DISEASE META-ANALYSES 25

SUPPLEMENT FIGURE 4: HEART FAILURE META-ANALYSES 26

SUPPLEMENTAL FIGURE 5: PERIOPERATIVE RISK REDUCTION META-ANALYSES 28

SUPPLEMENT FIGURE 6: HYPERTENSION META-ANALYSES 30

SUPPLEMENTAL FIGURE 7: HYPERTENSION META-ANALYSES ACCORDING TO BETA-BLOCKER TYPE 32

SUPPLEMENTAL REFERENCES 33

* Correspondence: d.kotecha@bham.ac.uk

Supplemental Methods

Search strategy

A systematic review of MEDLINE, EMBASE and the Cochrane Library were performed for randomised controlled trials (RCTs). The dates for this search were initially from inception of each database until April 2016, and then subsequently extended to December 2018. The search strategy included broad keywords and MeSH terms in four stages: (i) beta-blockers, including individual drug names; (ii) cardiovascular disease in general and also the specific named cardiovascular conditions; (iii) meta-analysis; and (iv) limitation to adults. We also manually searched reference lists of relevant studies, investigated registers of on-going trials and included studies after discussion with content experts.

MEDLINE	EMBASE	Cochrane
(i) beta-blockers		
exp adrenergic beta-antagonists/	exp beta adrenergic receptor blocking agent/	adrenergic beta-antagonists
beta blocker*.mp.	beta blocker*.mp.	beta blocker
beta receptor antagonist*.mp.	beta receptor antagonist*.mp.	beta receptor antagonist
acebutolol.mp.	acebutolol.mp.	acebutolol
atenolol.mp.	atenolol.mp.	atenolol
bisoprolol.mp.	bisoprolol.mp.	bisoprolol
bucindolol.mp.	bucindolol.mp.	bucindolol
carteolol.mp.	carteolol.mp.	carteolol
carvedilol.mp.	carvedilol.mp.	carvedilol
celiprolol.mp.	celiprolol.mp.	celiprolol
esmolol.mp.	esmolol.mp.	esmolol
labetalol.mp.	labetalol.mp.	labetalol
metoprolol.mp.	metoprolol.mp	metoprolol
nadolol.mp.	nadolol.mp.	nadolol
nebivolol.mp.	nebivolol.mp.	nebivolol
propanolol.mp.	propanolol.mp.	propranolol
(ii) cardiovascular disease		
exp cardiovascular diseases/	exp cardiovascular disease/	cardiovascular diseases
thoracic surgery/	exp cardiovascular surgery/	thoracic surgery
exp stroke/	exp cerebrovascular accident/	stroke
angina.mp.	angina.mp.	angina
heart failure.mp.	heart failure.mp.	heart failure
atrial fibrillation.mp.	atrial fibrillation.mp.	atrial fibrillation
myocardial infarction.mp.	myocardial infarction.mp.	myocardial infarction
acute coronary syndrome.mp.	acute coronary syndrome.mp.	acute coronary syndrome
hypertension.mp.	hypertension.mp.	hypertension
cardiac surgery.mp.	cardiac surgery.mp.	cardiac surgery
stroke*.mp.	stroke*.mp.	stroke
		prevention
		perioperative
(iii) meta-analysis		
meta-analysis/	meta analysis/	meta-analysis
meta-analysis as topic/	"meta analysis (topic)"/	meta-analysis as topic
(meta analy* or metaanaly*).mp.	(meta analy* or metaanaly*).mp.	(meta anlysis or metaanalysis)
(iv) limitation to adults		
limit to "all adult (19 plus years)"	limit to (adult 18 to 64 years or aged 65+ years)	limit to adult

Data collection, analysis and extraction of meta-analyses

Two investigators (OJZ, MS) independently examined the eligibility of all titles and abstracts of meta-analyses identified by the search strategy. Data were then independently extracted and tabulated in a standardised extraction form. Differences and missing data were resolved by group discussion, reference to the original publication and additional independent adjudication (DK).

All data were extracted from meta-analyses, including crude and adjusted outcome data where available. For coronary artery disease (CAD) trials were classified into acute myocardial infarction (MI) trials (if randomised within 48 hours of symptom onset) or non-acute trials (if >48 hours of symptoms), and by whether the majority of patients received reperfusion (pre-reperfusion trials if <50% of patients received reperfusion either with thrombolytics or coronary intervention, and reperfusion if ≥50%). In heart failure (HF), we assessed according to clinical subgroups: age, left ventricular ejection fraction (LVEF), heart rhythm and concomitant conditions. Perioperative studies were grouped by type of surgery (cardiac and non-cardiac) and risk of bias. Many meta-analyses for non-cardiac surgery include the Dutch Echocardiographic Cardiac Risk Evaluation Applying Stress Echocardiography (DECREASE) studies, which the sponsor declared as subject to potential scientific misconduct[1]; meta-analyses containing these studies were therefore defined as high risk. In hypertension, we considered different control groups (placebo, renin-angiotensin system [RAS] antagonists, calcium channels blockers [CCB] and diuretics), and performed a post-hoc sensitivity analysis according to beta-blocker agent (atenolol versus other beta-blockers).

Risk of bias and quality assessment

Two authors (DIB, JPH) independently assessed meta-analysis quality using the AMSTAR instrument (A Measurement Tool to Assess Multiple Systematic Reviews)[2] and the ROBIS tool (Risk of Bias in Systematic Reviews),[3] which address key criteria such as eligibility criteria, study identification and selection, study appraisal, data extraction and synthesis. Risk of bias in the individual RCTs was assessed with the Cochrane risk of bias tool.[4]

Additional statistical methods

Risk ratio (RR): Where the RR could not be calculated due to crude event data being unreported, we imputed the adjusted RR from the study, or converted the odds ratio to RR using published methods: RR = OR / ([1−pRef] + [pRef*OR]), where pRef is the prevalence of the outcome in the reference group.[5] There was insufficient reporting of hazard ratios to allow comparison across trials, and hence these were not used in analysis.

Supplemental Table 1: Details of included meta-analyses

INCLUDED STUDY	Population	Inclusion population definition	Primary outcome	Beta-blocker	Control	Studies n	Beta-blocker n	Control n	Follow-up (years)*	ROBIS Bias Risk	AMSTAR Quality score
					Coronary Artery Disease						
Al-Reesi 2008 [6]	Acute MI (not defined)	Randomised within 72 hours post MI	6 week mortality	Any	Placebo or no Tx	18	37,358	37,286	1.17	HIGH	6/11
Bangalore 2014 [7]	Acute MI (not defined)	RCTs >100 patients	All-cause mortality	Any	Placebo, no Tx or other Tx	60	15,004	20,642	9.77	HIGH	6/11
Brandler 2010 [8]	Acute coronary syndrome	Randomised within 24 hours post MI	In-hospital mortality	β1 antagonist	Placebo or no Tx	18	36,173	36,076	9.77	LOW	5/11
Chatterjee 2013 [9]	acute or suspected ACS with <48h onset	Randomised IV BB started <48hr after ACS onset.	In-hospital mortality	Any	Placebo or no Tx	16	36,737	36,659	0.69	LOW	7/11
Elgendy 2016[10]	STEMI undergoing PCI, Killip class 1 or 2	RCTs of IV BB vs placebo	All-cause mortality	Any	Placebo or no Tx	4	572	577	0.08	LOW	8/11
Freemantle 1999 [11]	Acute or past MI	RCTs without crossover. Tx >1day	All-cause mortality	Any	Placebo or no Tx	82	27,372	26,701	1.87	HIGH	8/11
Houghton 2000 [12]	HF post-MI	RCTs >50 patients. Started on BB or control post-MI on treatment for >1 month	All-cause mortality	Any	Placebo or no Tx	17	5408	5451	0.52	LOW	5/11
Huang HL 2012 [13]	Stable angina	RCTs, Treatment duration >3 weeks.	CV mortality	Any	Placebo	89	1186	1129	9.35	HIGH	3/11
Olsson 1992 [14]	Acute MI (not defined) with <48h onset	RCTs, placebo-controlled, metoprolol in acute treatment of MI	All-cause mortality	Metoprolol	Placebo	5	2753	2721	4.28	HIGH	3/11
Paladino 2010 [15]	STEMI given BB within 8hrs	RCTs, STEMI patients	In-hospital mortality	Any	Placebo or no Tx	18	36173	36076	0.62	LOW	2/11
Perez 2009[16]	ACS, LVF, dissection, stroke	Antihypertensive started within 24hrs of onset of acute event. Mortality data at 2 days, 10 days or >30days.	All-cause mortality	Any	Placebo or no Tx	65	9273	9208	0.79	LOW	10/11
Soriano 1997 [17]	Post-MI	RCT providing data on mortality. Published and unpublished data included	All-cause mortality	Any	Placebo	73	26036	25527	0.16	HIGH	4/11

INCLUDED STUDY	Population	Inclusion population definition	Primary outcome	Beta-blocker	Control	Studies n	Beta-blocker n	Control n	Follow-up (years)*	ROBIS Bias Risk	AMSTAR Quality score
					Heart Failure						
Abdulla 2006 [18]	Symptomatic HF. LVEF<45% on ACEi	RCT, Tx duration >12weeks	NYHA class and ETT	Any	Placebo	28	3727	3237	0.5	HIGH	6/11
Al-Gobari 2013 [19]	HF (EF not specified)	RCT, Tx duration >30 days with >3 months f/u	Sudden death	Any	Placebo, no Tx or other Tx	30	12768	12011	1	LOW	6/11
Azevum 1998 [20]	HFrEF	RCT	All-cause mortality	Any	Placebo	18	1606	1235	1.1	HIGH	2/11
Badve 2011 [21]	HFrEF and CKD	RCT, CKD stage 3-5, f/u >3 months. Reported mortality outcomes.	All-cause mortality	Any	Placebo, no Tx or other Tx	8	2868	2834	1	LOW	5/11
Bavishi 2014 [22]	HF with LVEF >40%	RCTs + prospective/retrospective cohort studies.	All-cause mortality	Any	Placebo	17	n/s	n/s	2.5	LOW	8/11
Bell 2006 [23]	HFrEF, 25% with diabetes	RCTs, placebo-controlled, Carvedilol as BB, HF due to LV systolic dysfunction.	All-cause mortality	Carved-ilol	Placebo	7	3034	2723	1	HIGH	4/11
Burnett 2017[24]	HFrEF	Network meta-analysis of medical therapies in HF	All-cause mortality	Any	Placebo	57	n/s	n/s	1	LOW	4/11
Bonet 2000 [25]	HF (EF not specified)	RCT parallel or crossover design, BB devoid of intrinsic sympathomimetic activity, Tx duration >8 weeks	All-cause mortality	Any	Placebo, no Tx or other Tx	21	3130	2719	0.5	HIGH	5/11
Bouzamond 2003 [26]	HF (EF not specified)	RCTs parallel design; data on mortality and hospitalisation outcomes	All-cause mortality, HF hospitalisation	Any	Placebo	16	7630	7227	1.2	HIGH	1/11
Brophy 2001 [27]	HF with LVEF <45%	RCT	All-cause mortality; HF hospitalisation	Any	Placebo	22	5273	4862	0.5	LOW	4/11
Cleland 2018[28]	IPD: HF with mean EF 27%	RCTs >300 patients, f/u >6 months, subgroup AF vs sinus rhythm, reported mortality, symptomatic HF	All-cause mortality	Any	Placebo	11	5,581	8,315	1.5	LOW	10/11
Cleophas 2001 [29]	HFrEF	RCT, mortality reported	All-cause mortality	Any	Placebo	4	3813	3679	1	UNCLEAR	2/11
Dulin 2005 [30]	HFrEF	RCT, subgroup >60 versus <60 years of age	All-cause mortality	Any	Placebo	5	n/s	n/s	1	HIGH	3/11
Fauchier 2007 [31]	HFrEF	RCT, subgroup ischaemic vs non-ischaemic aetiology. Mortality reported, f/u > 6months	All-cause mortality	Any	Placebo	8	3,792	3,458	1	LOW	4/11

INCLUDED STUDY	Population	Inclusion population definition	Primary outcome	Beta-blocker	Control	Studies n	Beta-blocker n	Control n	Follow-up (years)*	ROBIS Bias Risk	AMSTAR Quality score
Fukuta 2016 [32]	HFpEF	Compared observational studies and RCTs in HFpEF	All-cause mortality	Any	Placebo	3	519	527	2	LOW	9/11
Haas 2003 [33]	HFrEF	RCT >100 pts, subgroup diabetic vs non-diabetic, mortality outcome reported in diabetic subgroup	All-cause mortality	Any	Placebo	6	n/s	n/s	1.2	LOW	3/11
Heidenreich 1997 [34]	HFrEF	Parallel RCT, duration >3months, BB without intrinsic sympathomimetic activity, mortality reported	All-cause mortality	Any	Placebo	17	1723	1316	0.5	HIGH	3/11
Kotecha 2014[35]/ 2016[36]/ 2017[37]	IPD: HF with mean EF 27%	RCTs >300 patients, f/u >6 months, subgroup AF vs sinus rhythm, reported mortality, symptomatic HF	All-cause mortality	Any	Placebo	11	5,581	8,315	1.5	LOW	10/11
Krum 2005 [38]	HFrEF	RCT, >200 patients, reporting mortality, subgroup ACE/ARB vs no ACE/ARB and ACEi Tx duration ≤90/>90 days	All-cause mortality	Any	Placebo	12	6,843	6,527	1.4	LOW	4/11
Lechat 1998 [39]	HFrEF	RCT parallel design	All-cause mortality	Any	Placebo	18	1718	1305	0.6	HIGH	5/11
Lee 2001 [40]	HFrEF	RCT, reporting mortality	All-cause mortality	Any	Placebo	6	4735	4436	1.3	HIGH	4/11
Liu 2014 [41]	HF with LVEF >40%	RCT reporting mortality or hospitalisation outcomes, f/u>6months	All-cause mortality	Any	Placebo or no Tx	12	7834	13030	2.1	LOW	7/11
Martin 2018[42]	HF with LVEF >40%	RCTs with parallel group design enrolling adults	Cardiovascular mortality	Any	Placebo or no Tx	10	550	555	2.7	LOW	10/11
McAlister 2009 [43]	HFrEF	RCT reporting mortality	All-cause mortality	Any	Placebo	23	9820	9389	1	LOW	6/11
Nasr 2006 [44]	HFrEF	RCTs with parallel design, reporting AF incidence	Occurrence of new AF	Any	Placebo	7	6007	5944	1.4	LOW	6/11
O'Connor 2011 [45]	HFrEF	RCTs, primary endpoint of mortality. ITT analysis, Subgroup U.S.A. vs rest of world	All-cause mortality	Any	Placebo	4	5827	5808	1.5	LOW	4/11
Rienstra 2013 [46]	HF with LVEF <40%	RCTs, subgroup AF vs sinus rhythm, AF confirmed on ECG	All-cause mortality	Any	Placebo	4	4,482	4,198	0.75	LOW	7/11
Shekelle 2003 [47]	HFrEF	5 selected RCTs, >12 weeks duration	All-cause mortality	Any	Placebo	5	n/s	n/s	0.25	HIGH	4/11

INCLUDED STUDY	Population	Inclusion population definition	Primary outcome	Beta-blocker	Control	Studies n	Beta-blocker n	Control n	Follow-up (years)*	ROBIS Bias Risk	AMSTAR Quality score
Shibata 2001 [48]	HFrEF	Published parallel RCTs	All-cause mortality	Any	Placebo or no Tx	22	5507	4973	0.9	HIGH	4/11
Van Veldhuisen 2013 [49]	HF with LVEF >40%	RCTs, patients on ACEi + diuretics	All-cause mortality	Any	Placebo	3	519	529	2.7	HIGH	5/11
Wali 2011 [50]	HFrEF	RCT, subgroup CKD vs no CKD	All-cause mortality	Carvedil ol	Placebo	2	2,115	2,102	1.1	HIGH	3/11
Whorlow 2000 [51]	HFrEF NYHA class 4	Published RCT, patients on ACEi, diuretics ± digoxin. Tx duration >3 months	All-cause mortality	Any	Placebo	18	313	322	0.75	LOW	3/11
Zaman 2017 [52]	All HF	RCTs calculating excess mortality from deferring medical therapy for 1 year	All-cause mortality	Any	Placebo	21	n/s	n/s	11.73	LOW	5/11
Perioperative											
Angeli 2010 [53]	Non-cardiac surgery	RCTs reporting mortality	All-cause mortality	Any	Placebo	9	5274	5270	0.076	LOW	10/11
Angeli 2010 [54]	Non-cardiac surgery	RCTs reporting CV and all-cause mortality	CV mortality; all-cause mortality	Any	Placebo, no Tx or other Tx	24	6623	6325	0.076	UNCLEAR	3/11
Arsenault 2013 [55]	Cardiac-surgery	RCTs, no history of chronic AF	Post-op AF or SVT	Any	Placebo, no Tx or other Tx	33	2294	2404	0.058	LOW	11/11
Badgett 2010 [56]	Cardiac-surgery	Revised Cardiac Index of ≥1. BB administered before induction of anaesthesia and continued post-op	Total mortality; stroke during hospitalisation	Any	Placebo, no Tx or other Tx	7	5457	5455	n/s	LOW	5/11
Bangalore 2008 [57]	Non-cardiac surgery	RCTs, BB started in peri-op period, ±CV comorbidities, assessed outcomes within 30 days of surgery	20-day ACM, CV mortality, non-fatal MI, non-fatal stroke, HF	Any	Placebo, no Tx or other Tx	33	6311	5995	0.066	LOW	10/11
Biccard 2008 [58]	Non-cardiac surgery	Selected studies from five recent systematic reviews reporting either CV mortality or non-fatal MI.	CV mortality, non-fatal MI at 30 days	Any	Placebo	8	976	955	0.083	LOW	5/11
Blessberger 2014 [59]	Any surgery	RCTs, subgroup cardiac vs non-cardiac surgery. >70% under GA. Peri-op period is ±30 days	All-cause mortality	Any	Placebo, no Tx or other Tx	89	7769	7477	0.083	LOW	11/11
Bouri 2014 [60]	Non-cardiac surgery	BB initiated in pre-op period.	All-cause mortality	Any	Placebo	9	5264	5265	0.083	LOW	9/11

INCLUDED STUDY	Population	Inclusion population definition	Primary outcome	Beta-blocker	Control	Studies n	Beta-blocker n	Control n	Follow-up (years)*	ROBIS Bias Risk	AMSTAR Quality score
Dai 2014 [61]	Non-cardiac surgery	RCTs, ≥1 risk-factor for CAD, reported ACM, MI or stroke	ACM, MI ± stroke	Any	Placebo	8	5457	5723	0.17	HIGH	7/11
Devereaux 2005 [62]	Non-cardiac surgery	RCTs	All-cause mortality, adverse effects	Any	Placebo	4	453	454	0.046	LOW	8/11
Guay 2013 [63]	Any surgery	RCT, reported mortality at 30 days and 1yr.	All-cause mortality	Any	Placebo	12	5550	5551	0.20	HIGH	7/11
Ji 2016 [64]	CABG	RCTs reporting new-onset	New-onset AF	Any	Placebo	13	1158	1199	n/s	HIGH	8/11
Khan 2013 [65]	Cardiac surgery	RCTs reporting AF or SVT	AF or SVT	Any	Placebo or no Tx	10	1280	1276	n/s	HIGH	9/11
Landoni 2010 [66]	Non-cardiac surgery	RCTs, no restriction to dose/time of administration.	AF or SVT	Esmolol	Placebo or no Tx	32	853	912	n/s	HIGH	6/11
McGory 2005 [67]	Non-cardiac surgery	RCTs, started BB preoperatively, evaluation ≥1 relevant outcome	Perioperative + long-term all-cause mortality	Any	Placebo	8	354	278	0.15	HIGH	9/11
Mostafaie 2015 [68]	Non-cardiac surgery	RCTs non-cardiac vascular surgery, initiated BB preoperatively	All-cause mortality, CV mortality	Any	Placebo or no Tx	2	301	298	0.083	LOW	11/11
Sakamoto 2014 [69]	Cardiac-surgery	RCTs in Japanese patients	Post-operative AF	Landiol ol	Placebo, no Tx or other Tx	6	302	258	0.019	HIGH	9/11
Schouten 2005 [70]	Non-cardiac surgery	RCTs reporting ≥ 1 of perioperative MI and cardiac mortality	Perioperative MI, cardiac mortality	Any	Placebo or no Tx	15	551	526	0.020	HIGH	8/11
Talati 2009 [71]	Non-cardiac surgery	RCTs in BB naïve patients initiated preoperatively	All-cause mortality, MI, stroke	Any	Placebo	6	5094	5089	0.34	LOW	7/11
Wang 2013 [72]	Cardiac-surgery	RCTs reporting post-operative AF	Post-operative AF	Carvedil ol	Placebo, no Tx or other Tx	2	111	102	n/s	LOW	6/11
Weisbauer 2007 [73]	Any surgery	RCTs, BB initiated pre/intraoperative or 1 day post surgery. Subgroup cardiac vs. non-cardiac surgery	All-cause mortality, adverse effects	Any	Placebo or no Tx	21	2206	2198	0.083	LOW	11/11
Wijeysunde ra 2014 [74]	Non-cardiac surgery	RCTs or cohort studies >100 patients, BB started ≤45 days prior to surgery or ≤24hrs post.	All-cause mortality, MI, CV mortality, stroke	Any	Placebo or no Tx	16	5986	5977	0.26	LOW	11/11

INCLUDED STUDY	Population	Inclusion population definition	Primary outcome	Beta-blocker	Control	Studies n	Beta-blocker n	Control n	Follow-up (years)*	ROBIS Bias Risk	AMSTAR Quality score
Zangrillo 2009 [75]	Cardiac-surgery	RCTs, no restriction in dose and timing of BB.	Myocardial ischaemia; arrhythmias	Esmolol	Placebo or no Tx	20	386	392	n/s	HIGH	9/11
					Hypertension						
Balamuthusamy 2009 [76]	Diabetic with HTN	RCTs in diabetic hypertension	MI, stroke, CV mortality, total mortality.	Any	Other Tx	8	5072	5281	5.4	HIGH	**7**/11
Bangalore 2007 [77]	HTN	Follow up >/= 1 yr. RCTs with randomised comparisons of regimens based on BB v other agents	New-onset DM	Any	Placebo, no Tx or other Tx	12	n/s	n/s	4	HIGH	**5**/11
Bangalore Cardio-protection 2008 [78]	HTN	RCTs. BB used as first-line treatment for HTN, f/u >1 year. Reported cardiovascular outcomes	ACM, CV mortality, MI, Stroke, HF	Any	Placebo, no Tx or other Tx	9	34096	34124	3.5	LOW	**7**/11
Bangalore Prevention 2008 [79]	HTN	RCTs, hypertension with cardiovascular RFs but no established HF. BB as first line monotherapy, f/u >1 yr. HF reported as outcome	New-onset HF.	Any	Other Tx	6	52,857	13,665	3.5	LOW	8/11
Bradley 2006 [80]	HTN	RCTs with BB as first-line drug or monotherapy	All-cause mortality	Any	Placebo or no Tx	4	9109	14504	5	HIGH	11/11
Carlberg 2004 [81]	HTN	RCTs in primary hypertension, treatment with atenolol as monotherapy and first-line drug	ACM, CV mortality	Atenolol	Placebo or no Tx	8	2625	3767	4.6	LOW	6/11
Cruickshank 2017[82]	HTN <60yrs	4 meta-analyses investingating obesity, sympathetic hyperactivity and beta blockers	Mortality, stroke, MI	Any	Placebo or no Tx	n/s	n/s	n/s	n/s	HIGH	3/11
De Lima Luiz 2014 [83]	HTN with prior stroke or TIA	RCT, clinical outcomes	Stroke recurrence	Atenolol	Placebo or no Tx	2	1104	1089	3	LOW	11/11
Ding 2012 [84]	HTN	RCT, f/u > 2 years, sample size of >100 patients	Non-fatal and fatal stroke	Any	Other Tx	5	n/s	n/s	3	LOW	5/11
Jeffers 2016 [85]	HTN with prior stroke or CAD	CCB vs other antihythertensive agents on cardiovascular outcomes	Mortality, MI, stroke	Any	Placebo or no Tx	3	n/s	n/s	3	LOW	8/11

INCLUDED STUDY	Population	Inclusion population definition	Primary outcome	Beta-blocker	Control	Studies n	Beta-blocker n	Control n	Follow-up (years)*	ROBIS Bias Risk	AMSTAR Quality score
Khan 2006 [86]	HTN	RCTs comparing BB as first line for HTN in preventing major cardiovascular outcomes. Subgroup >60yrs vs <60yrs	Composite of: Stroke (non-fatal); MI (non-fatal); CV death	Any	Placebo	2	7588	11826	3	HIGH	6/11
Kuyper 2014 [87]	HTN	RCTs using BBs as first-line in hypertension. Subgroup: Atenolol	ACM, Stroke, MI, composite CV outcomes	Atenolol	Placebo or no Tx	4	11,025	16,408	n/s	HIGH	5/11
Law 2009 [88]	HTN	RCT	CHD, stroke	Any	Placebo		n/s	n/s	n/s	HIGH	4/11
Lindholm 2005 [89]	HTN	RCT of primary HTN, BB as first line antihypertensive in at least 50% of pts	ACM, CV mortality	Any	Placebo or no Tx	7	11025	16408	n/s	LOW	4/11
Messerli 1998 [90]	HTN >60yrs	RCTs, Tx duration > least 1 year, used diuretics and/or BB as first-line. Elderly cohort >/= 60 years	Stroke + TIA), Stroke mortality, CV mortality, ACM.	Any	Placebo or no Tx	10	1521	2678	n/s	HIGH	4/11
Palla 2017 [91]	HTN black patients	RAS inhibitors vs other antihypertensive agents on cardiovascular outcomes	Mortality, MI, stroke	Any	Placebo	3	3376	3377	2	LOW	8/11
Psaty 1997 [92]	HTN	RCTs, vascular disease, f/u >1 year	Stroke, CHD, CHF, mortality	Any	Placebo	4	383	700	1.5	LOW	8/11
Remonti 2016[93]	HTN with type 2 diabetes	MA of RCTs of antihypertensive agents	All-cause mortality	Any	Placebo or no Tx	30	n/s	n/s	3	LOW	9/11
Sciarretta 2011 [94]	HTN with high CV risk	RCTs, high CV risk and >65% of pts with HTN, sample size >200. Reported absolute incidence	New onset HF	Any	Other Tx	3	14564	14644	4.3	LOW	8/11
Shinton 1990 [95]	HTN	RCT, reported mortality, cerebrovascular and CHD events	All-cause mortality	Any	Placebo, no Tx or other Tx	3	11858	11826	n/s	UNCLEAR	3/11
Venkata 2010 [96]	HTN	RCT, subgroup atenolol vs non-atenolol	Incident stroke	Any	Other Tx	12	51963	53882	n/s	UNCLEAR	1/11
Wang 2016[97]	HTN with prior stroke	Bayesian network MA of antihypertensive agents on reducing stroke, CHD, MACCE	Stroke	Any	Placebo or no Tx	2	1104	1104	2.6	LOW	6/11
Wiysonge 2012[98]	HTN	RCT, Tx duration >1 year, BB as monotherapy or first-line drug	All-cause mortality	Any	Placebo	4	n/s	n/s	n/s	LOW	11/11
Wiysonge 2017[99]	HTN	RCT, Tx duration >1 year, BB as monotherapy or first-line drug	All-cause mortality	Any	Placebo	4	n/s	n/s	n/s	LOW	11/11

INCLUDED STUDY	Population	Inclusion population definition	Primary outcome	Beta-blocker	Control	Studies n	Beta-blocker n	Control n	Follow-up (years)*	ROBIS Bias Risk	AMSTAR Quality score
Wright 1999 [100]	HTN	RCT, Tx duration >1 year, defined end-points, >70% in treatment group still taking drug after 1 year	ACM, stroke, CAD, Sudden cardiac death, total CV events	Any	Placebo or no Tx	2	5505	10867	n/s	HIGH	6/11
Wright 2000 [101]	HTN	RCT, BB or thiazides as first line therapy	All-cause mortality	Any	Placebo	2	5505	10867	n/s	UNCLEAR	2/11
Wright 2009 [102]	HTN	RCTs, Tx duration >1 year, reported ITT analysis	ACM, stroke, CV events, withdrawal due to adverse effects	Any	Placebo or no Tx	5	6967	12346	4.5	LOW	11/11
Xue 2015 [103]	HTN	RCTs with parallel design, > 6 months f/u, primary hypertension, Reported morbidity or mortality	ACM, MI, Stroke, HF hospitalisation, ESRF	Any	Other Tx	2	4611	4628	4.8	LOW	11/11

*as provided or weighted calculation based on number of participants; ACM, all-cause mortality; AF, atrial fibrillation; AMSTAR, A Measurement Tool to Assess Systematic Reviews; BB, beta-blocker; CABG, coronary artery bypass graft; CKD, chronic kidney disease; CV, cardiovascular; ETT, exercise tolerance test; f/u, follow-up; HF, heart failure; LVEF, left ventricular ejection fraction; LVF, left ventricular failure; MI, myocardial infarction; n/s, not stated; NYHA, New York Heart Association; RCT, randomised controlled trial; ROBIS, Risk of Bias in Systematic Reviews; STEMI; ST elevation myocardial infarction; SVT, supraventricular tachycardia; Tx, treatment.

<u>**Supplemental Table 2: Details of excluded meta-analyses**</u>

Excluded study	Inclusion population definition	Exclusion Reason	Sample size	Results
Coronary artery disease				
Heidenreich 1999 [104]	Randomised studies comparing BB, CCB and long acting nitrates.	Did not compare BB vs placebo/no treatment	90 RCTs involving 72 BB vs CCB and 6 BB vs nitrates	Cardiac mortality and MI were not significantly different between BB vs CCB. Fewer episodes of angina and adverse events with BB than CCB
Howes 1995 [105]	Meta-analysis of atenolol, celiprolol, enalapril, nifedipine and doxazocin on cholesterol and BP	Did not report clinical outcomes	23 RCTs involving 15 on Atenolol and 5 on Celiprolol.	Atenolol reduced HDL-C and increased total cholesterol, LDL-C and triglycerides compared with others
Huang 2015 [106]	Meta-analysis of observational studies assessing beta-blockers in patients with MI undergoing PCI	Observational studies included	10 studies involving 40,873 patients	Beta-blockers were associated with reduced mortality (adjusted HR .76, 95% CI 0.62-0.94) but not with CV mortality, recurrent MI or HF hospitalisation
Jia 2015 [107]	Meta-analysis of RCTs assessing Tongxinluo capsule vs BBs in patients with angina	No hard clinical outcomes reported	73 RCTs including 7424 patients	Tongxinluo improved symptoms and ECG improvements significantly more than BBs
Misumida 2015 [108]	Observational studies assessing beta-blockers in STEMI patients undergoing PCI with EF >40%	Observational studies included	7 observational studies involving 10,857 patients	Beta-blockers were associated with reduced mortality HR 0.79, 95% CI 0.65-0.97
Shu 2012 [109]	Diagnosed or suspected IHD. RCTs with parallel design, sub-grouped into placebo and no Txt comparison	Did not perform systematic search and formal meta-analysis	2 studies (1 placebo, 1 no Tx).	All-cause mortality: no Tx comparison OR 0.40 95% CI 0.20-0.79; placebo comparison OR 0.92, 95% CI 0.62-1.38
Heart Failure				
Briasoulis 2015 [110]	HFrEF patients. Compared carvedilol vs metoprolol	Did not compare BB vs placebo/no treatment	10 studies. 30,943 on carvedilol and 69,925 on metoprolol. Follow up 36.4 months.	Mortality was reduced with carvedilol vs metoprolol in prospective studies only. No difference in hospitalisation
Chatterjee 2013 [111]	Compared different BB in HF patients	Did not compare BB vs placebo/no treatment	21 trials. 23,122 patients	No differences between BB in mortality

Excluded study	Inclusion population definition	Exclusion Reason	Sample size	Results
DiNicolantonia 2013 [112]	Compared different BBs in setting of AMI or systolic HF	Did not compare BB vs placebo/no treatment	8 trials of 4,563 patients	In both AMI and HF trials, cardvedilol significantly reduced mortality compared to placebo/ no treatment
Dobre 2007 [113]	Systematic review of efficacy and tolerability of BB in elderly patients with HF	Does not separate BB from other antihypertensive agents in a meta-analysis	3 trials	BB are well tolerate and effective in elderly HF patients
Leizorovicz 2002 [114]	Meta-analysis of RCTs comparing bisoprolol vs placebo	Not a systematic approach to search	2 RCTs (CIBIS and CIBIS II) including 3288 patients	Bisoprolol reduced mortality and hospitalisation compared to placebo
Packer 2001 [115]	Meta-analysis of RCTs comparing carvedilol with metoprolol	Did not report hard clinical end-points (only LVEF change). Compared BB vs BB.	19 RCTs	Carvedilol increased LVEF more than metoprolol
Prins 2015 [116]	Meta-analysis of observational and randomised studies	Compared BB withdrawal vs BB continuation	5 observational and 1 randomised study including 2,704 continued on BB and 439 discontinued	Discontinuation of BBs in acute decompensated HF significantly increased mortality and rehospitalisation
Zarembski 1996 [117]	Meta-analysis of RCTs assessing BB versus placebo in dilated cardiomyopathy	Only reports NYHA class and LVEF change	11 RCTs including 623 patients	Low dose BB improved NYHA functional class and LVEF compared to placebo
Perioperative				
Crystal 2002 [118]	RCTs in CABG ± valve surgery. Reported SVT incidence	Did not provide relevant clinical outcomes	27 trials	Reduced incidence of AF and SVT
DiNicolantonia 2014 [119]	Compared carvedilol vs metoprolol on incidence of AF in CABG	Did not compare BB vs placebo/no treatment	4 trials of 601 patients.	Carvedilol significantly reduced post-operative AF compared to metoprolol.
Kaw 2011 [120]	Meta-analysis of studies evaluating the association of new onset AF after CABG with mortality	Compared patients with AF vs non-AF (and not BB vs control)	11 RCTs including 40,112 patients	Perioperative BB reduced occurrence of AF whereas ACEi increased it
Ollila 2018[121]	Meta-analysis of RCTs evaluating intraperoperative BB use in mon-cardiac surgery	Not a meta-analysis of all-cause mortality: only 1 RCT included.	2 RCTs including 133 patients	Esmolol reduced myocardial ischaemia but had no significant evet on composite of cardiac events, hypotension or mortality

Excluded study	Inclusion population definition	Exclusion Reason	Sample size	Results
Yu 2011 [122]	Non-cardiac surgery, esmolol v control, studies provide details on dose/infusion protocols.	Does not provide crude numbers so risk ratio outcome cannot be calculated.	67 RCTs	Esmolol reduced myocardial ischaemia (OR 0.17, 95% CI 0.02-0.45). Increased incidence of hypotension (dose related) but not bradycardia with esmolol
Hypertension				
Aursnes 2003 [123]	Bayesian fixed effect model	Included studies did not separate diuretic therapy from beta-blockers	27 trials	BB or diuretics are similar to ACEi and CCB in stroke and HF prevention but superior to CCB in preventing coronary events
Baguet 2005 [124]	Calculation of the sum weighted for trial size	Did not report clinical outcomes	72 RCTs comprising 9,094 patients	SBP reduction more marked with diuretics, CCB and ACEi. Drug classes had a similar magnitude of effect on DBP
Baguet 2007 [125]	Mean BP reduction for drug classes evaluated by combing data and weighting by trial size.	Did not report clinical outcomes	80 RCTs involving 10,818 patients	Atenolol reduced SBP by 15.2mmHg and DBP by 12.1mmHg. Largest SBP reduction seen with CCB and ACEi whilst DBP were generally similar between classes but most marked with BB
Dahlof 2007 [126]	RCT, first-line BB, Tx duration >1 year or >1000 patient years of f/u	Did not report crude data, only report HR for stroke.	5 RCTs including 12537 subjects	Beta blocker based antihypertensive therapy reduced cardiovascular risk compared to placebo or no treatment
Germino 2012 [127]	Nebivolol vs placebo in 12 week RCT. Pooled changes in BP and heart rate and adverse events	Did not report clinical outcomes	3 RCTs involving 1380 on nebivolol and 205 on placebo.	Nebivolol significantly reduced both SBP and DBP compared to placebo, but with less efficacy in patients >62 years. Similar rates of adverse events between groups
Marpillat 2013 [127]	Network meta-analysis of antihypertensive therapy on cognition	Only outcome reported is cognitive decline	19 RCTs (n = 18,515) and 11 studies (n = 831,674)	BBs were less effective at reducing cognitive decline compared to ARBs, but not compared to CCBs, ACEi and diuretics
Magee 1999 [128]	Meta-analysis of RCTs investigating BBs in pregnancy hypertension	Did not report relevant clinical outcomes	34 RCTs	BBs were associated with an increase in small for gestational age, ›ut decreased severe hypertension, proteinuria and respiratory distress syndrome
Mulrow 2009 [129]	Cochrane review of RCTs of >1 year duration in hypertensive elders (≥60 years)	Did not separate BBs from other antihypertensive therapies	15 RCTs including 24,055 subjects	Antihypertensive therapy reduced mortality in those 60 years or older but not those 80 years or older
Psaty 200 3[130]	RCTs, f/u >1 year, network meta-analysis comparing to low dose diuretics only	Not systematic BB vs control	42 RCTs including 192,478 subjects	Low dose diuretics were the most effective first line treatment to prevent cardiovascular morbidity and mortality.

Excluded study	Inclusion population definition	Exclusion Reason	Sample size	Results
Turnbull 200 3[131]	Meta-analysis of effects of different antihypertensive therapies on clinical outcomes	Did not separate diuretic therapy from beta-blockers	29 RCTs including 162,341 patients	There were no differences in major cardiovascular events between ACEi, CCB or diuretics/BB, although ACEi reduced BP less
Turnbull 2005 [132]	Meta-analysis of BP lowering regimens in patients with and without diabetes	Did not separate diuretic therapy from beta-blockers	27 RCTs including 158,709 patients	Major CV events were reduced similarly in those with and without diabetes by ACEi, CCB, ARB and diuretics/BB

ACEi, angiotensin converting enzyme inhibitor; AF, atrial fibrillation; ARB, angiotensin receptor blocker; BB, beta blocker; AMI, acute myocardial infarction; BP, blood pressure; CABG, coronary artery bypass graft; CCB, calcium channel blocker; CI, confidence interval; CIBIS, Cardiac Insufficiency Bisoprolol Study; CV, cardiovascular; DBP, diastolic blood pressure; f/u, follow up; HDL, high density lipoprotein; HF, heart failure; HR, hazard ratio; LDL, low density lipoprotein; LVEF, left ventricular ejection fraction; MI, myocardial infarction; NYHA, New York Heart Association; OR, odds ratio; PCI, percutaneous coronary intervention; RCTs, randomised controlled trials; SBP, systolic blood pressure; STEMI, ST-elevation myocardial infarction; SVT, supraventricular tachycardia.

Supplemental Table 3: ROBIS results for each individual meta-analysis

The ROBIS checklist tool was used to assess any concerns with the review process, including study eligibility criteria, identification and selection of studies, data collection and study appraisal, and synthesis of findings. The overall risk of bias is the interpretation of review findings, and whether these considered the limitations found in the domains above.

Study	Comment	Study eligibility criteria	Identification and selection of studies	Data collection and study appraisal	Synthesis and findings	Risk of bias in the review
Coronary Artery Disease						
Al-Reesi 2008[6]		Low	Low	Low	Low	High
Bangalore 2014[7]		Low	Low	Low	Low	Low
Brandler 2010[8]		Low	Low	Low	High	Low
Chatterjee 2013[9]		Low	Low	Low	Low	Low
Elgendy 2016[10]		Low	Low	High	Low	Low
Freemantle 1999[11]		Low	Low	Low	High	High
Houghton 2000[12]		Low	High	High	High	Low
Huang HL 2012[13]		Low	Low	High	High	High
Olsson 1992[14]		High	High	Low	High	High
Paladino 2010[15]		Low	Low	High	High	Low
Perez 2009[16]	Registered	Low	Low	Low	Low	Low
Shu 2012[109]		Low	High	High	High	High
Soriano 1997[17]		Low	Low	Low	Low	High
Heart Failure						
Abdulla 2006[18]		Low	Low	High	Low	High
Al-Gobari 2013[19]		Low	High	High	Low	Low
Azevum 1998[20]		Low	High	Low	Low	High
Badve 2011[21]		High	High	High	Low	Low
Bavishi 2014[22]		Low	Low	Low	Low	Low
Bell 2006[23]		Low	High	High	High	High
Bonet 2000[25]		Low	Low	High	High	High
Bouzamondo 2003[26]		Low	High	High	High	High
Burnett 2017[24]		Low	Low	High	Low	Low
Brophy 2001[27]		Low	High	High	Low	Low
Cleland 2018[28]	Registered IPD	Low	Low	Low	Low	Low

Cleophas 2001[29]		Low	High	High	High	Unclear
Dulin 2005[30]		Low	High	High	High	High
Fauchier 2007[31]		Low	High	High	Low	Low
Fukuta 2016[32]		Low	Low	High	Low	Low
Haas 2003[33]		Low	High	High	Low	Low
Heidenreich 1997[34]		Low	Low	High	High	High
Kotecha 2014[35]	Registered IPD	Low	Low	Low	Low	Low
Kotecha 2016[36]	Registered IPD	Low	Low	Low	Low	Low
Kotecha 2017[28]	Registered IPD	Low	Low	Low	Low	Low
Krum 2005[38]		Low	Low	High	High	Low
Lechat 1998[39]		Low	High	High	Low	High
Lee 2001[40]		Low	High	High	Low	High
Liu 2014[41]		Low	High	Low	Low	Low
Martin 2018[42]		Low	Low	Low	Low	Low
McAlister 2009[43]		Low	Low	Low	Low	Low
Nasr 2006[44]		Low	Low	Low	Low	Low
O'Connor 2011[45]		Low	High	High	Low	Low
Rienstra 2013[46]		Low	Low	Low	Low	Low
Shekelle 2003[47]		High	High	High	Low	High
Shibata 2001[48]		High	Low	High	High	High
Van Veldhuisen 2013[46]		High	High	High	High	High
Wali 2011[50]		Low	Low	Low	Low	High
Whorlow 2000[51]		Low	High	High	High	Low
Zaman 2017[52]		Low	Low	High	Low	Low
Perioperative						
Angeli 2010[53] bias		Low	Low	Low	Low	Low
Angeli 2010[54] mortality		High	High	High	High	Unclear
Arsenault 2013[55]		Low	Low	Low	Low	Low
Badgett 2010[56]		Low	High	High	Low	Low
Bangalore 2008[57]		Low	Low	Low	Low	Low
Biccard 2008[58]		High	High	High	Low	Low
Blessberger 2014[59]	Registered	Low	Low	Low	Low	Low
Bouri 2014[60]		Low	Low	Low	Low	Low

Dai 2014[61]		Low	Low	Low	Low	High
Devereauz 2005[62]		Low	Low	Low	Low	Low
Guay 2013[63]		Low	Low	Low	Low	High
Ji 2016[64]		Low	Low	Low	Low	High
Khan 2013[65]		Low	Low	Low	Low	High
Landoni 2010[66]		Low	Low	Low	Low	High
McGory 2005[67]		Low	Low	Low	Low	High
Mostafaie 2015[68]	Registered	Low	Low	Low	Low	Low
Sakamoto 2014[69]		Low	Low	Low	Low	High
Schouten 2005[133]		Low	Low	Low	High	High
Talati 2009[71]		Low	Low	High	Low	Low
Wang 2013[72]		Low	Low	Low	Low	Low
Weisbauer 2007[73]		Low	Low	Low	Low	Low
Wijeysundera 2014[74]		Low	Low	Low	Low	Low
Zangrillo 2009[75]		Low	Low	Low	Low	High
Hypertension						
Balamuthusamy 2009[76]		Low	Low	Low	Low	High
Bangalore 2007[77]		Low	High	High	Low	High
Bangalore 2008[78] Cardioprotection		Low	Low	High	Low	Low
Bangalore 2008[79] Prevention		Low	Low	Low	Low	Low
Bradley 2006[80]		Low	Low	Low	Low	High
Carlberg 2004[81]		Low	Low	High	High	Low
Cruickshank 2017[82]		Low	High	High	High	High
De Lima Luiz 2014[83]	Registered	Low	Low	Low	Low	Low
Ding 2012[84]		Low	Low	High	High	Low
Jeffers 2016[85]		Low	Low	Low	Low	Low
Khan 2006[86]		Low	Low	Low	Low	High
Kuyper 2014[87]		Low	Low	High	Low	High
Law 2009[88]		Low	Low	Low	Low	High
Lindholm 2005[89]		Low	Low	High	High	Low
Messerli 1998[90]		Low	Low	High	Low	High
Palla 2017[91]		High	Low	Low	Low	Low

Psaty 1997[92]		Low	High	Low	Low	Low
Remonti 2016[93]		Low	Low	Low	Low	Low
Sciarretta 2011[94]		Low	Low	Low	Low	Low
Shinton 1990[95]		Low	High	High	High	Unclear
Venkata 2010[96]		Low	Unclear	Unclear	Unclear	Unclear
Wang 2016[134]		Low	High	Low	Low	Low
Wiysonge 2012[98]		Low	Low	Low	Low	Low
Wiysonge 2017[99]		Low	Low	Low	Low	Low
Wright 1999[100]		Low	Low	Low	High	High
Wright 2000[101]		High	High	High	High	Unclear
Wright 2009[102]	Registered	Low	Low	Low	Low	Low
Xue 2015[103]	Registered	Low	Low	Low	Low	Low

Registered indicates the review was prospectively registered with a publicly available database, for example in PROSPERO. For references see Supplement 2. IPD, individual patient-data meta-analysis.

Supplemental Table 4: GRADE Scale for Assessment of Certainty of Evidence

The Grading of Recommendations, Assessment, Development and Evaluation (GRADE) approach tool was used to assess the certainty of evidence. It provides a structured and transparent evaluation of the importance of outcomes, using a comprehensive criteria for downgrading or upgrading the certainty of evidence based on five factors: risk of bias, inconsistency of results, indirectness of evidence, imprecision in effect estimates, and publication bias. The overall certainty of evidence is the combined rating of the quality of evidence across these factors.

Study	Risk of bias	Inconsistency	Indirectness	Imprecision	Publication bias	Overall certainty of evidence
Coronary Artery Disease: Acute Coronary Syndrome (trials after routine reperfusion)						
All-cause mortality	Not serious	Serious	Not serious	Not serious	None	Moderate
Incident myocardial infarction	Not serious	Not serious	Not serious	Not serious	None	High
Incident stroke	Not serious	Not serious	Not serious	Very serious	None	Low
Incident heart failure	Not serious	Not serious	Not serious	Serious	None	Moderate
Coronary Artery Disease: Acute Coronary Syndrome (trials before routine reperfusion)						
All-cause mortality	Not serious	Serious	Not serious	Not serious	None	Moderate
Incident myocardial infarction	Not serious	Not serious	Not serious	Not serious	None	High
Incident stroke	Not serious	Not serious	Not serious	Very serious	None	Low
Incident heart failure	Not serious	Not serious	Not serious	Serious	None	Moderate
Coronary Artery Disease: Non-acute ischaemic heart disease (trials after routine reperfusion)						
All-cause mortality	Not serious	Serious	Not serious	Very serious	None	Very low
Incident myocardial infarction	Not serious	Serious	Not serious	Very serious	None	Very low
Incident stroke	Not serious	Not serious	Not serious	Very serious	None	Low
Incident heart failure	Not serious	Not serious	Not serious	Not serious	None	High
Coronary Artery Disease: Non-acute ischaemic heart disease (trials before routine reperfusion)						
All-cause mortality	Not serious	Not serious	Not serious	Not serious	None	High
Incident myocardial infarction	Not serious	Not serious	Not serious	Not serious	None	High
Incident stroke	Not serious	Not serious	Not serious	Very serious	None	Low
Incident heart failure	Not serious	Not serious	Not serious	Serious	None	Moderate
Heart failure with LVEF < 40%, in sinus rhythm						
All-cause mortality	Not serious	Not serious	Not serious	Not serious	None	High
Cardiovascular mortality	Not serious	Not serious	Not serious	Not serious	None	High

Heart failure hospitalisation	Not serious	Not serious	Not serious	Not serious	None	High
Incident stroke	Not serious	Not serious	Not serious	Very serious	None	Low
Heart failure with LVEF < 40%, in atrial fibrillation						
All-cause mortality	Not serious	Not serious	Not serious	Not serious	None	High
Cardiovascular mortality	Not serious	Not serious	Not serious	Not serious	None	High
Heart failure hospitalisation	Not serious	Not serious	Not serious	Not serious	None	High
Incident stroke	Not serious	Not serious	Not serious	Very serious	None	Low
Heart failure with LVEF ≥ 40%						
All-cause mortality	Not serious	Serious	Not serious	Serious	None	Moderate
Cardiovascular mortality	Not serious	Serious	Not serious	Serious	None	Low
Heart failure hospitalisation	Not serious	Serious	Not serious	Serious	None	Low
Perioperative: Non-cardiac surgery (high risk of bias trials)						
All-cause mortality	Very serious	Not serious	Very serious	Serious	High	Very low
Incident myocardial infarction	Very serious	Not serious	Very serious	Not serious	High	Very low
Incident stroke	Very serious	Serious	Serious	Very serious	High	Very low
Perioperative: Non-cardiac surgery (low risk of bias trials)						
All-cause mortality	Not serious	Not serious	Not serious	Serious	None	Moderate
Incident myocardial infarction	Not serious	Not serious	Not serious	Not serious	None	High
Incident stroke	Not serious	Not serious	Not serious	Not serious	None	High
Perioperative: Cardiac surgery						
All-cause mortality	Not serious	Not serious	Not serious	Very serious	None	Low
Incident myocardial infarction	Not serious	Not serious	Not serious	Very serious	None	Low
Incident stroke	Not serious	Not serious	Not serious	Very serious	None	Low
Hypertension: Beta-blocker vs placebo						
All-cause mortality	Not serious	Not serious	Not serious	Serious	None	Moderate
Incident myocardial infarction	Not serious	Not serious	Not serious	Serious	None	Moderate
Incident stroke	Not serious	Serious	Not serious	Serious	None	Low
Hypertension: Beta-blocker vs Diuretic						
All-cause mortality	Not serious	Not serious	Not serious	Serious	None	Moderate

Incident myocardial infarction	Not serious	Not serious	Not serious	Very serious	None	Low
Incident stroke	Not serious	Not serious	Not serious	Very serious	None	Low
Hypertension: Beta-blocker vs Renin angiotensin system antagonist						
All-cause mortality	Not serious	Not serious	Not serious	Serious	None	Moderate
Incident myocardial infarction	Not serious	Not serious	Not serious	Not serious	None	High
Incident stroke	Not serious	Not serious	Not serious	Not serious	None	High
Hypertension: Beta-blocker vs Calcium channel blocker						
All-cause mortality	Not serious	Not serious	Not serious	Serious	None	Moderate
Incident myocardial infarction	Not serious	Not serious	Not serious	Not serious	None	High
Incident stroke	Not serious	Not serious	Not serious	Not serious	None	High

Risk of bias was considered serious if the risk reduces confidence in the estimated treatment effect. Risk of bias was considered very serious if the risk is sufficiently large that the confidence in the estimated treatment effect is considerably lower. Inconsistency was considered serious if analyses do not share a consistent treatment effect. Inconsistency was considered very serious if analyses had dissimilar point estimates, non-overlapping confidence intervals, and significant heterogeneity. Indirectness was considered serious if cumulative evidence was derived from trials assessing interventions in participants with varying baseline cardiovascular risk. Indirectness was considered very serious if cumulative evidence was derived from trials assessing interventions in participants with wide variety of sub-indications. Serious imprecision was considered if the 95% confidence intervals overlaps with the minimally important difference for clinical benefit (RR >1.10). Very serious imprecision was considered if the 95% confidence intervals include both clinically important benefit (RR <0.90) and harm (RR >1.10).

Supplemental Figure 1. PRISMA flow diagram

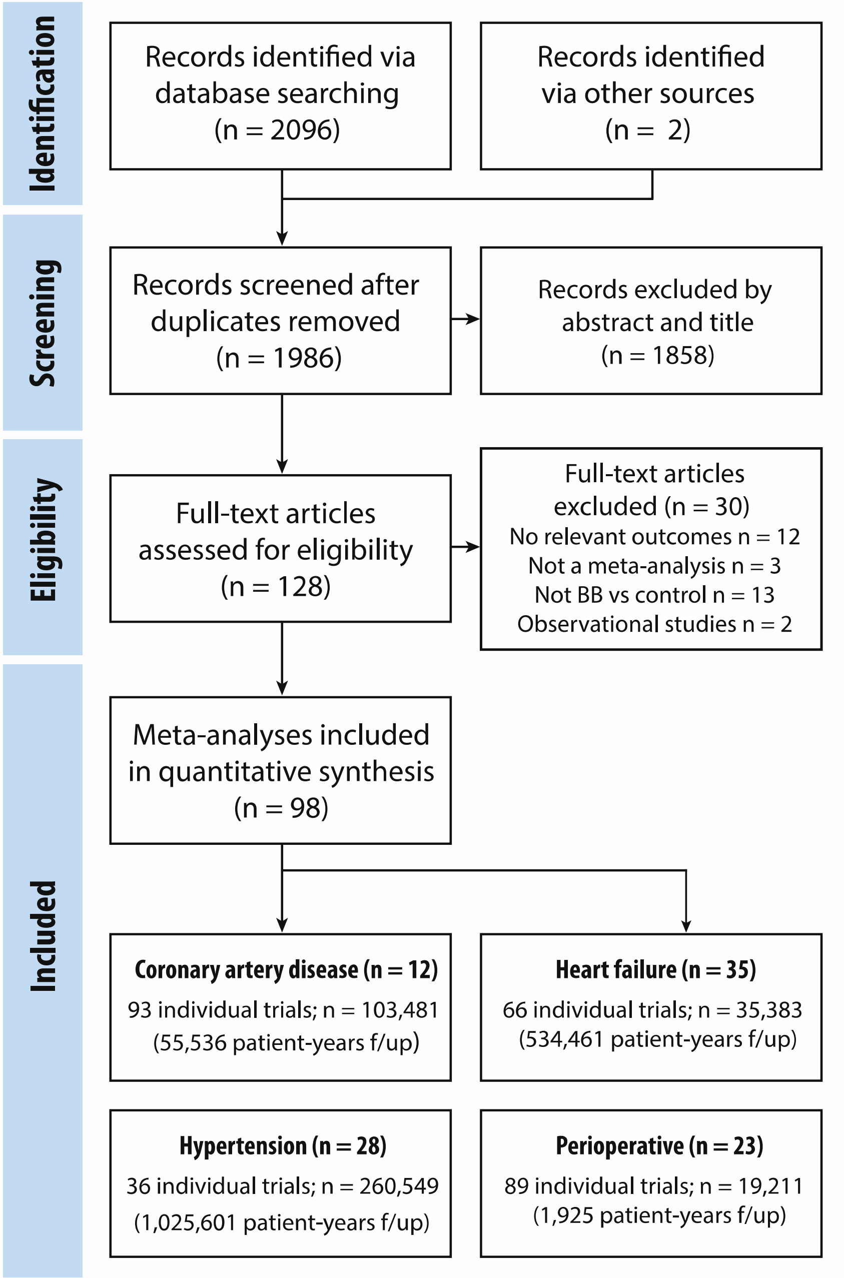

Numbers (n) reflect the number of included or excluded meta-analyses in our systematic review.
BB, beta-blocker; f/up, follow-up.

Supplemental Figure 2. ROBIS results from meta-analyses in each cardiovascular condition

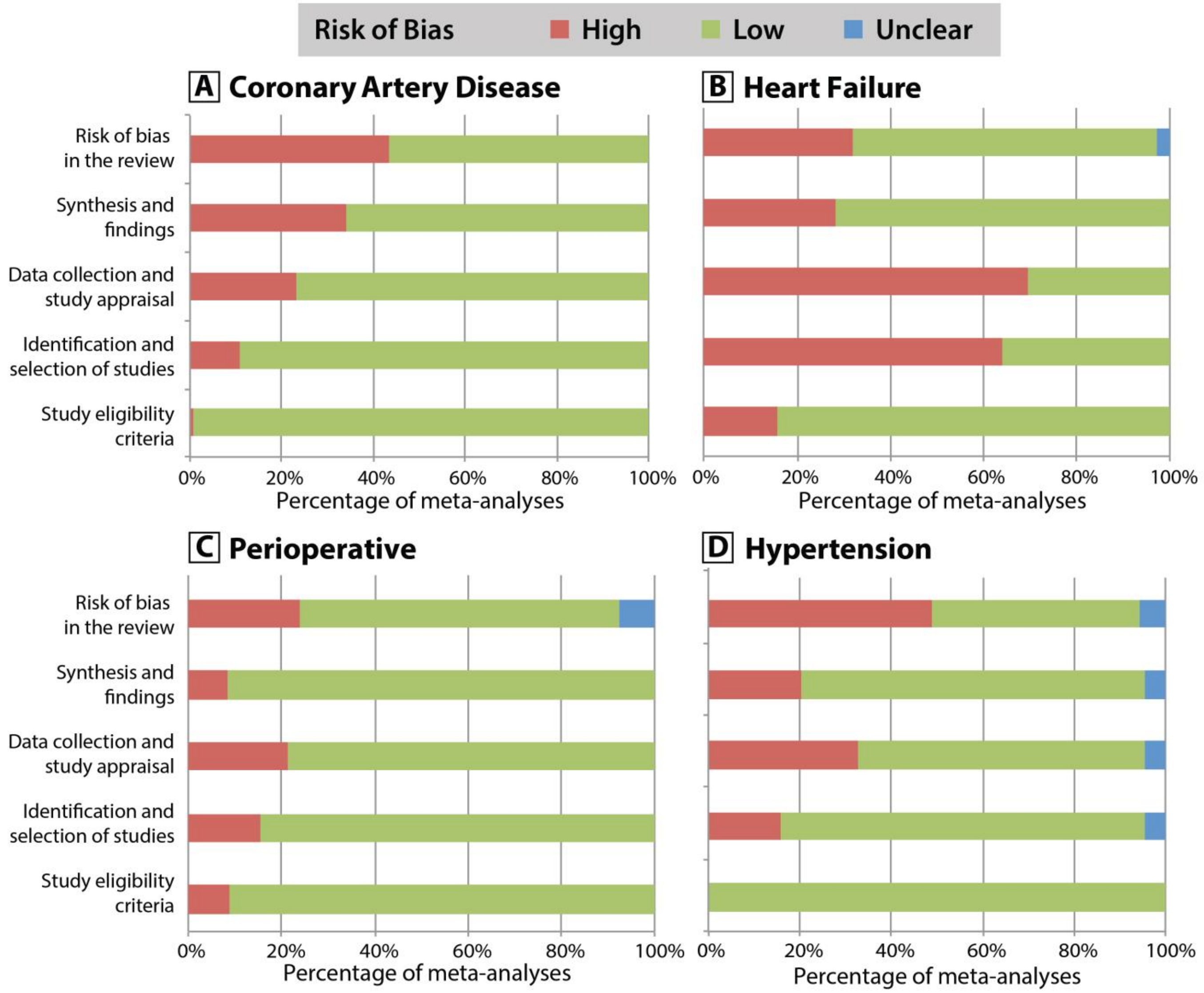

Graphical presentation of the ROBIS (Risk Of Bias In Systematic review) checklist results from all included meta-analyses categorised into each cardiovascular condition. Red colour represents high risk of bias, green represents low risk of bias, and blue represents unclear bias risk. The "risk of bias in review" category indicates the overall risk of bias rating.

Supplemental Figure 3: Coronary artery disease meta-analyses

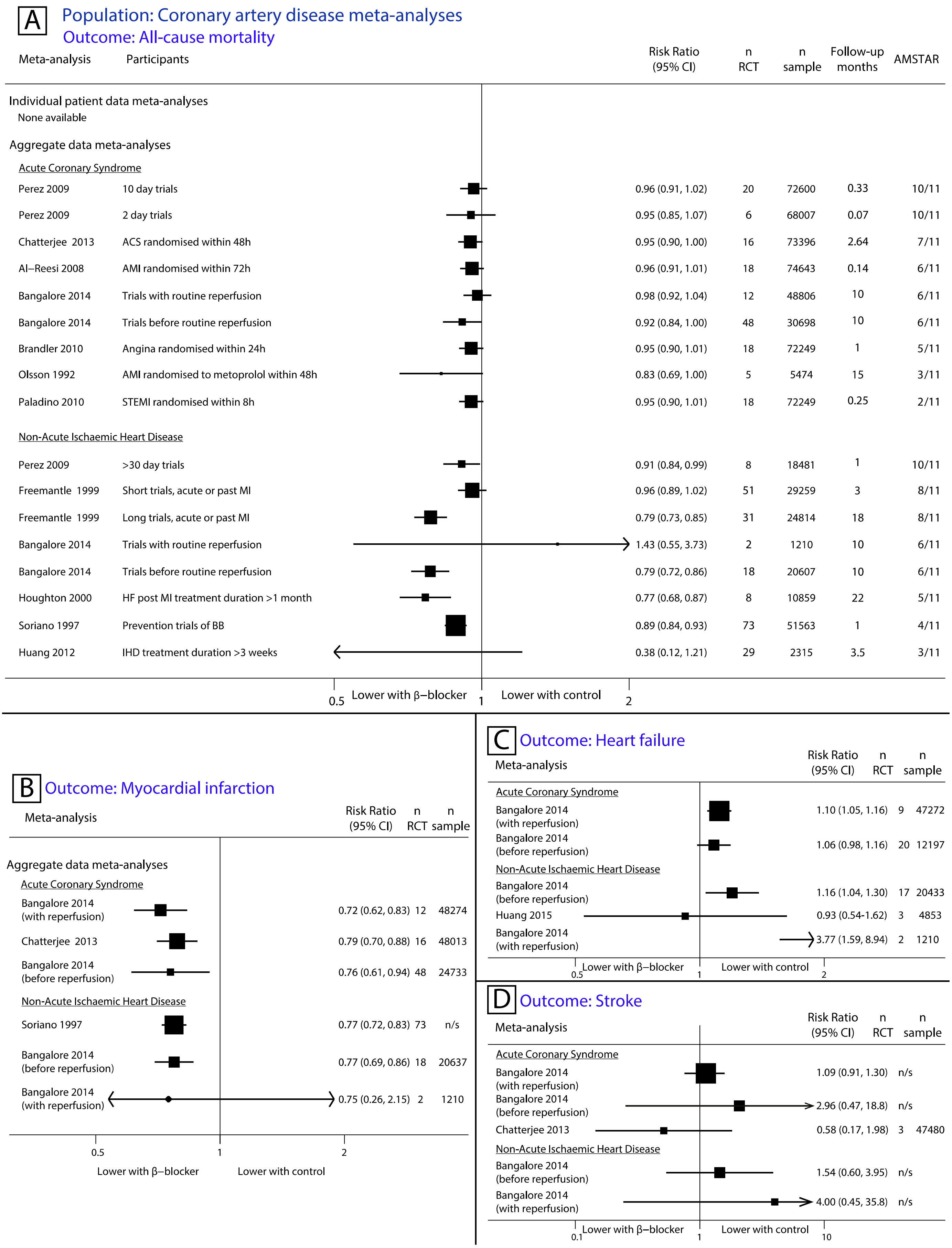

Summary plots of meta-analyses for coronary artery disease, including A) all-cause mortality; B) myocardial infarction; and C) heart failure; ordered by study quality using the AMSTAR index.

Supplement Figure 4: Heart failure meta-analyses

A Population: Heart failure meta-analyses

Outcome: All-cause mortality

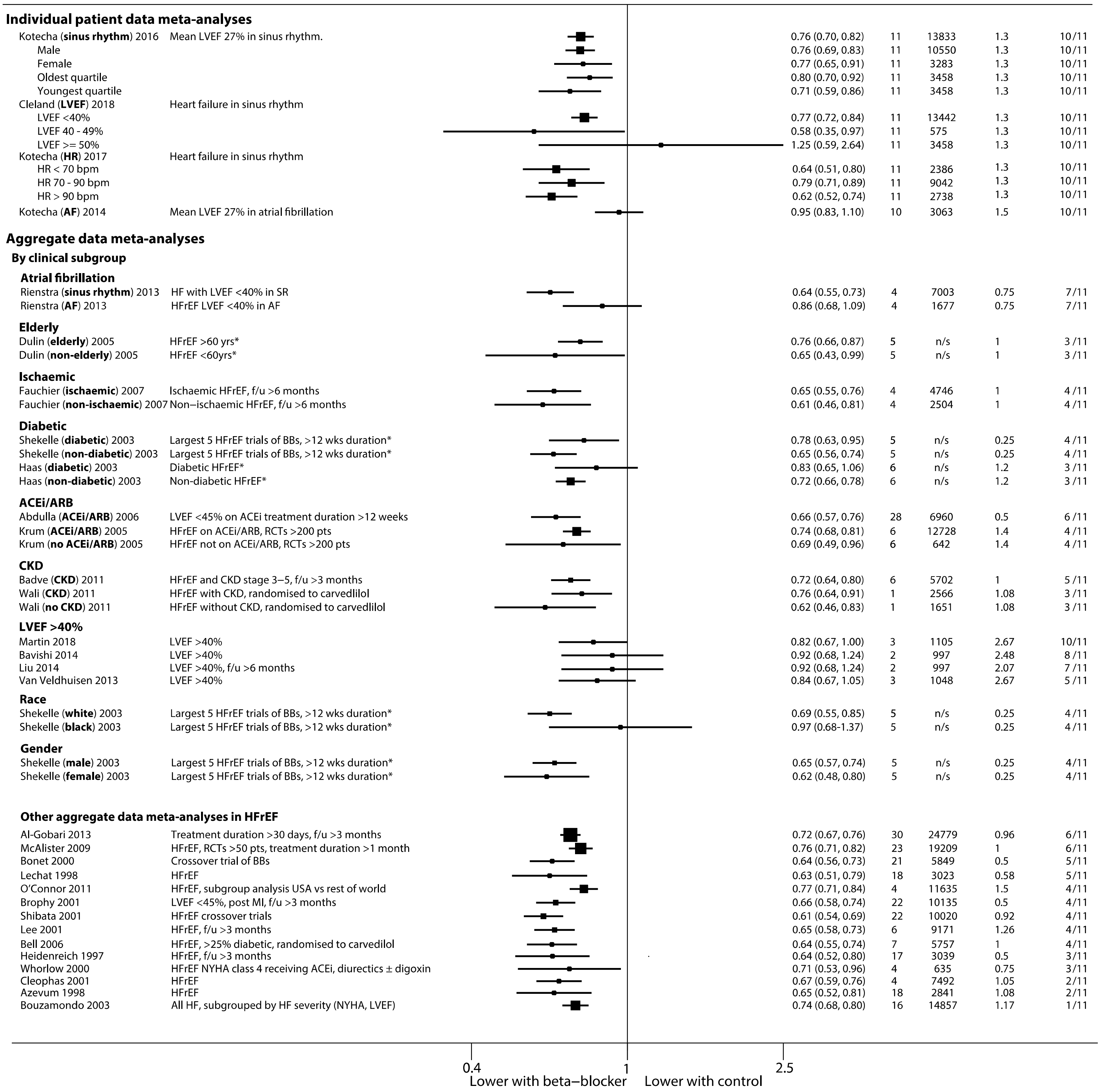

Meta-analysis	Participants	Risk Ratio (95% CI)	n RCT	n sample	Follow-up years	AMSTAR
**Individual patient data meta-analyses**						
Kotecha (**sinus rhythm**) 2016	Mean LVEF 27% in sinus rhythm.	0.76 (0.70, 0.82)	11	13833	1.3	10/11
Male		0.76 (0.69, 0.83)	11	10550	1.3	10/11
Female		0.77 (0.65, 0.91)	11	3283	1.3	10/11
Oldest quartile		0.80 (0.70, 0.92)	11	3458	1.3	10/11
Youngest quartile		0.71 (0.59, 0.86)	11	3458	1.3	10/11
Cleland (**LVEF**) 2018	Heart failure in sinus rhythm					
LVEF <40%		0.77 (0.72, 0.84)	11	13442	1.3	10/11
LVEF 40 - 49%		0.58 (0.35, 0.97)	11	575	1.3	10/11
LVEF >= 50%		1.25 (0.59, 2.64)	11	3458	1.3	10/11
Kotecha (**HR**) 2017	Heart failure in sinus rhythm					
HR < 70 bpm		0.64 (0.51, 0.80)	11	2386	1.3	10/11
HR 70 - 90 bpm		0.79 (0.71, 0.89)	11	9042	1.3	10/11
HR > 90 bpm		0.62 (0.52, 0.74)	11	2738	1.3	10/11
Kotecha (**AF**) 2014	Mean LVEF 27% in atrial fibrillation	0.95 (0.83, 1.10)	10	3063	1.5	10/11
**Aggregate data meta-analyses**						
**By clinical subgroup**						
**Atrial fibrillation**						
Rienstra (**sinus rhythm**) 2013	HF with LVEF <40% in SR	0.64 (0.55, 0.73)	4	7003	0.75	7/11
Rienstra (**AF**) 2013	HFrEF LVEF <40% in AF	0.86 (0.68, 1.09)	4	1677	0.75	7/11
**Elderly**						
Dulin (**elderly**) 2005	HFrEF >60 yrs*	0.76 (0.66, 0.87)	5	n/s	1	3 /11
Dulin (**non-elderly**) 2005	HFrEF <60yrs*	0.65 (0.43, 0.99)	5	n/s	1	3 /11
**Ischaemic**						
Fauchier (**ischaemic**) 2007	Ischaemic HFrEF, f/u >6 months	0.65 (0.55, 0.76)	4	4746	1	4 /11
Fauchier (**non-ischaemic**) 2007	Non–ischaemic HFrEF, f/u >6 months	0.61 (0.46, 0.81)	4	2504	1	4 /11
**Diabetic**						
Shekelle (**diabetic**) 2003	Largest 5 HFrEF trials of BBs, >12 wks duration*	0.78 (0.63, 0.95)	5	n/s	0.25	4 /11
Shekelle (**non-diabetic**) 2003	Largest 5 HFrEF trials of BBs, >12 wks duration*	0.65 (0.56, 0.74)	5	n/s	0.25	4 /11
Haas (**diabetic**) 2003	Diabetic HFrEF*	0.83 (0.65, 1.06)	6	n/s	1.2	3 /11
Haas (**non-diabetic**) 2003	Non-diabetic HFrEF*	0.72 (0.66, 0.78)	6	n/s	1.2	3 /11
**ACEi/ARB**						
Abdulla (**ACEi/ARB**) 2006	LVEF <45% on ACEi treatment duration >12 weeks	0.66 (0.57, 0.76)	28	6960	0.5	6 /11
Krum (**ACEi/ARB**) 2005	HFrEF on ACEi/ARB, RCTs >200 pts	0.74 (0.68, 0.81)	6	12728	1.4	4 /11
Krum (**no ACEi/ARB**) 2005	HFrEF not on ACEi/ARB, RCTs >200 pts	0.69 (0.49, 0.96)	6	642	1.4	4 /11
**CKD**						
Badve (**CKD**) 2011	HFrEF and CKD stage 3–5, f/u >3 months	0.72 (0.64, 0.80)	6	5702	1	5 /11
Wali (**CKD**) 2011	HFrEF with CKD, randomised to carvedlilol	0.76 (0.64, 0.91)	1	2566	1.08	3 /11
Wali (**no CKD**) 2011	HFrEF without CKD, randomised to carvedlilol	0.62 (0.46, 0.83)	1	1651	1.08	3 /11
**LVEF >40%**						
Martin 2018	LVEF >40%	0.82 (0.67, 1.00)	3	1105	2.67	10/11
Bavishi 2014	LVEF >40%	0.92 (0.68, 1.24)	2	997	2.48	8 /11
Liu 2014	LVEF >40%, f/u >6 months	0.92 (0.68, 1.24)	2	997	2.07	7 /11
Van Veldhuisen 2013	LVEF >40%	0.84 (0.67, 1.05)	3	1048	2.67	5 /11
**Race**						
Shekelle (**white**) 2003	Largest 5 HFrEF trials of BBs, >12 wks duration*	0.69 (0.55, 0.85)	5	n/s	0.25	4 /11
Shekelle (**black**) 2003	Largest 5 HFrEF trials of BBs, >12 wks duration*	0.97 (0.68-1.37)	5	n/s	0.25	4 /11
**Gender**						
Shekelle (**male**) 2003	Largest 5 HFrEF trials of BBs, >12 wks duration*	0.65 (0.57, 0.74)	5	n/s	0.25	4/11
Shekelle (**female**) 2003	Largest 5 HFrEF trials of BBs, >12 wks duration*	0.62 (0.48, 0.80)	5	n/s	0.25	4/11
**Other aggregate data meta-analyses in HFrEF**						
Al-Gobari 2013	Treatment duration >30 days, f/u >3 months	0.72 (0.67, 0.76)	30	24779	0.96	6/11
McAlister 2009	HFrEF, RCTs >50 pts, treatment duration >1 month	0.76 (0.71, 0.82)	23	19209	1	6/11
Bonet 2000	Crossover trial of BBs	0.64 (0.56, 0.73)	21	5849	0.5	5/11
Lechat 1998	HFrEF	0.63 (0.51, 0.79)	18	3023	0.58	5/11
O'Connor 2011	HFrEF, subgroup analysis USA vs rest of world	0.77 (0.71, 0.84)	4	11635	1.5	4/11
Brophy 2001	LVEF <45%, post MI, f/u >3 months	0.66 (0.58, 0.74)	22	10135	0.5	4/11
Shibata 2001	HFrEF crossover trials	0.61 (0.54, 0.69)	22	10020	0.92	4/11
Lee 2001	HFrEF, f/u >3 months	0.65 (0.58, 0.73)	6	9171	1.26	4/11
Bell 2006	HFrEF, >25% diabetic, randomised to carvedilol	0.64 (0.55, 0.74)	7	5757	1	4/11
Heidenreich 1997	HFrEF, f/u >3 months	0.64 (0.52, 0.80)	17	3039	0.5	3/11
Whorlow 2000	HFrEF NYHA class 4 receiving ACEi, diurectics ± digoxin	0.71 (0.53, 0.96)	4	635	0.75	3/11
Cleophas 2001	HFrEF	0.67 (0.59, 0.76)	4	7492	1.05	2/11
Azevum 1998	HFrEF	0.65 (0.52, 0.81)	18	2841	1.08	2/11
Bouzamondo 2003	All HF, subgrouped by HF severity (NYHA, LVEF)	0.74 (0.68, 0.80)	16	14857	1.17	1/11

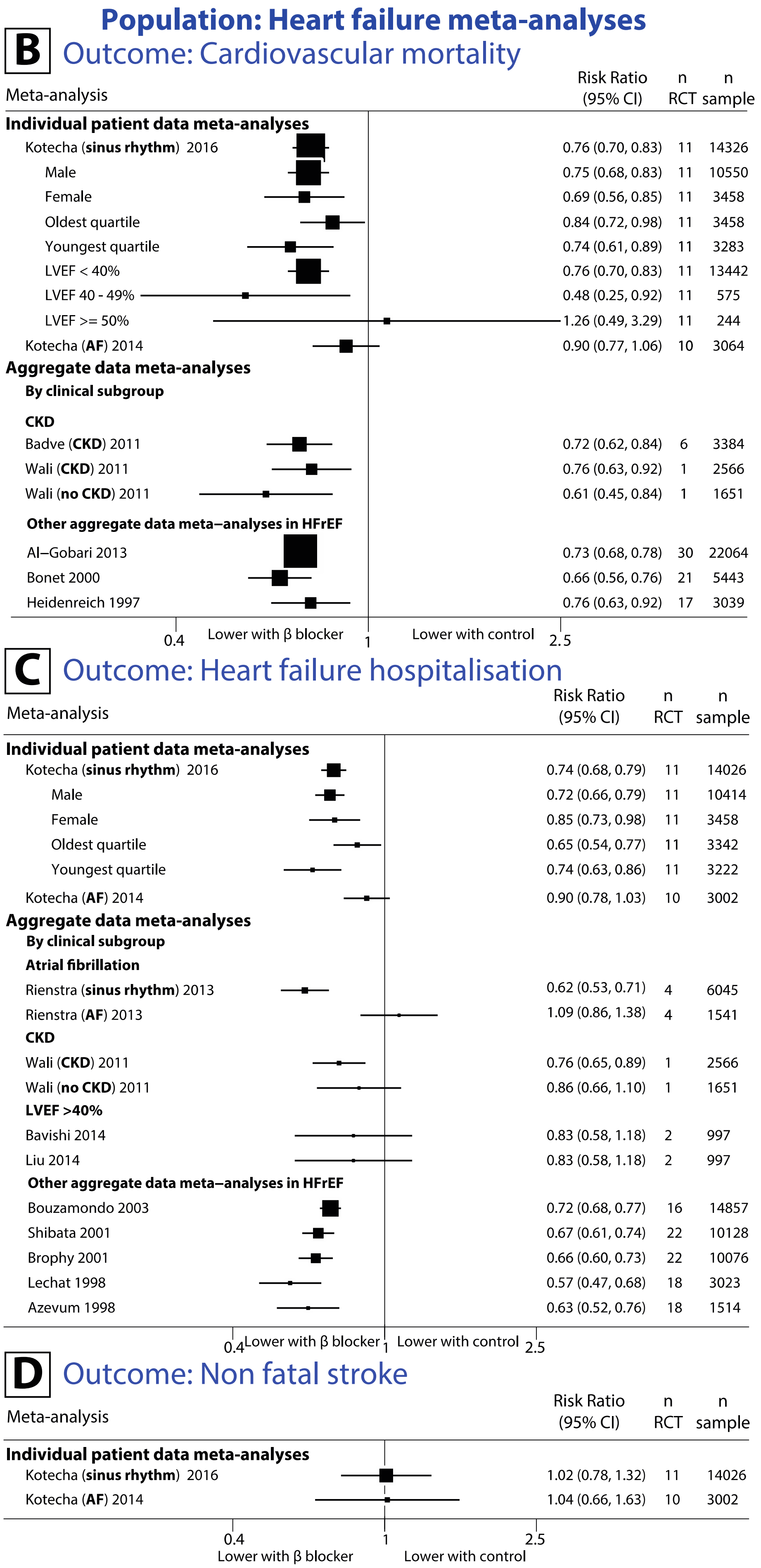

Summary of meta-analyses for heart failure reporting A) all-cause mortality; B) cardiovascular mortality; C) heart failure hospitalisation; and D) non-fatal stroke; ordered by AMSTAR. * adjusted outcome.

Supplemental Figure 5: Perioperative risk reduction meta-analyses

A Population: Perioperative meta-analyses

Outcome: All-cause mortality

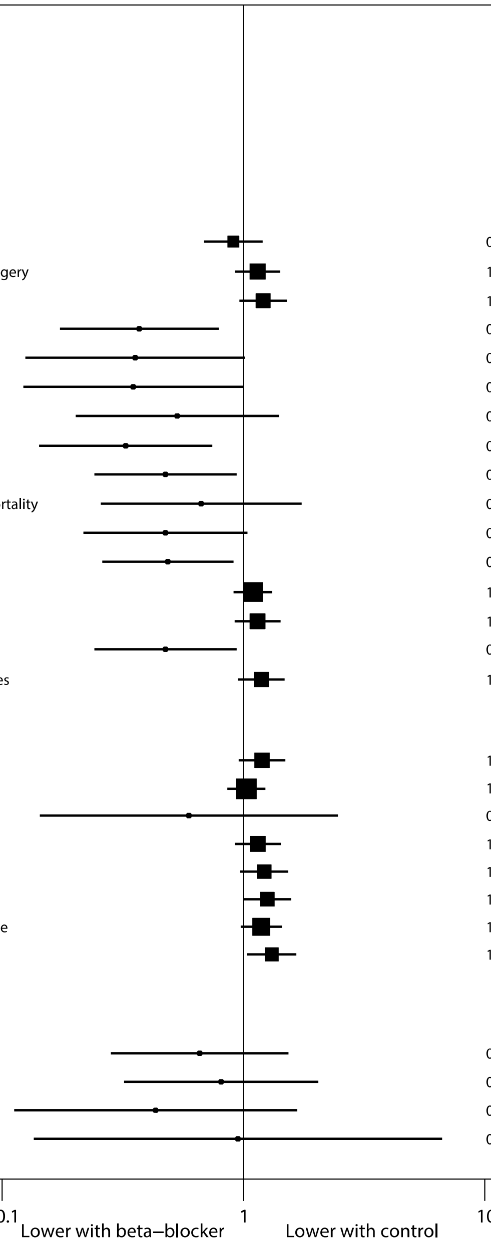

Meta-analysis	Participants	Risk Ratio (95% CI)	n RCT	n sample	Follow-up years	AMSTAR
**Individual patient data meta-analyses**						
None available						
**Aggregate data meta-analyses**						
**Non-cardiac surgery**						
**High risk of bias**						
Weisbauer 2007	BB perioperative	0.91 (0.69, 1.20)	8	2057	0.08	11/11
Wijeysundera 2014	BB given 45 days prior to 24h after surgery	1.14 (0.92, 1.42)	16	11963	0.26	11/11
Angeli 2010	Intermediate risk surgery	1.21 (0.96, 1.51)	19	12134	0.08	10/11
Angeli 2010	High risk surgery	0.37 (0.17, 0.79)	5	794	0.08	10/11
Angeli 2010	High risk of bias	0.36 (0.12, 1.01)	10	788	0.08	10/11
Bangalore 2008	High bias, perioperative BB	0.35 (0.12, 1.00)	10	748	0.07	10/11
McGory 2005	Short term trials, perioperative BB	0.53 (0.20, 1.40)	2	312	0.02	9/11
McGory 2005	Long term trials, perioperative BB	0.32 (0.14, 0.74)	2	312	1	9/11
Bouri 2014	Non-secure trials, preoperative BB	0.48 (0.24, 0.94)	2	1178	0.23	9/11
Schouten 2005	Report perioperative MI or cardiac mortality	0.67 (0.26, 1.74)	15	1077	0.02	8/11
Devereaux 2005	Report safety and efficacy	0.47 (0.22, 1.04)	4	907	0.05	8/11
Guay 2013	Report outcomes at 1 year	0.49 (0.26, 0.91)	4	781	1	7/11
Dai 2014	>1 risk factor for CAD	1.09 (0.91, 1.31)	8	11180	0.17	7/11
Guay 2013	Report outcomes at 30 days	1.14 (0.92, 1.42)	12	11101	0.08	7/11
Badgett 2010	High bias, revised cardiac index of >0	0.48 (0.24, 0.94)	5	1178	n/s	5/11
Angeli 2010	Cardiovascular and mortality outcomes	1.19 (0.95, 1.48)	24	10544	0.08	3/11
**Low risk of bias**						
Blessberger 2014	30 day trials, cochrane review	1.19 (0.95, 1.49)	36	11463	0.08	11/11
Blessberger 2014	Long term trials, cochrane review	1.03 (0.86, 1.23)	36	11463	1	11/11
Mostafaie 2015	Only non-cardiac vascular surgery	0.59 (0.14, 2.46)	2	599	0.08	11/11
Angeli 2010	Low risk of bias	1.15 (0.92, 1.43)	14	12140	0.08	10/11
Bangalore 2008	Perioperative BB	1.22 (0.97, 1.54)	13	11114	0.07	10/11
Bouri 2014	Secure trials, preoperative BB	1.26 (1.00, 1.58)	7	10529	0.23	9/11
Talati 2009	BB naïve patients treated perioperative	1.19 (0.97, 1.44)	6	10183	0.34	7/11
Badgett 2010	Revised cardiac index of >0	1.31 (1.04, 1.66)	2	9734	n/s	5/11
**Cardiac surgery**						
Blessberger 2014	Cardiac surgery, cochrane review	0.66 (0.28, 1.54)	53	3783	0.08	11/11
Arsenault 2013	CABG or valvular surgery, no prior AF	0.81 (0.32, 2.04)	33	2671	0.06	11/11
Weisbauer 2007	Cardiac surgery, perioperative BB	0.43 (0.11, 1.67)	13	2347	0.08	11/11
Zangrillo 2009	Esmolol vs control/placebo	0.95 (0.14, 6.65)	20	302	n/s	9/11

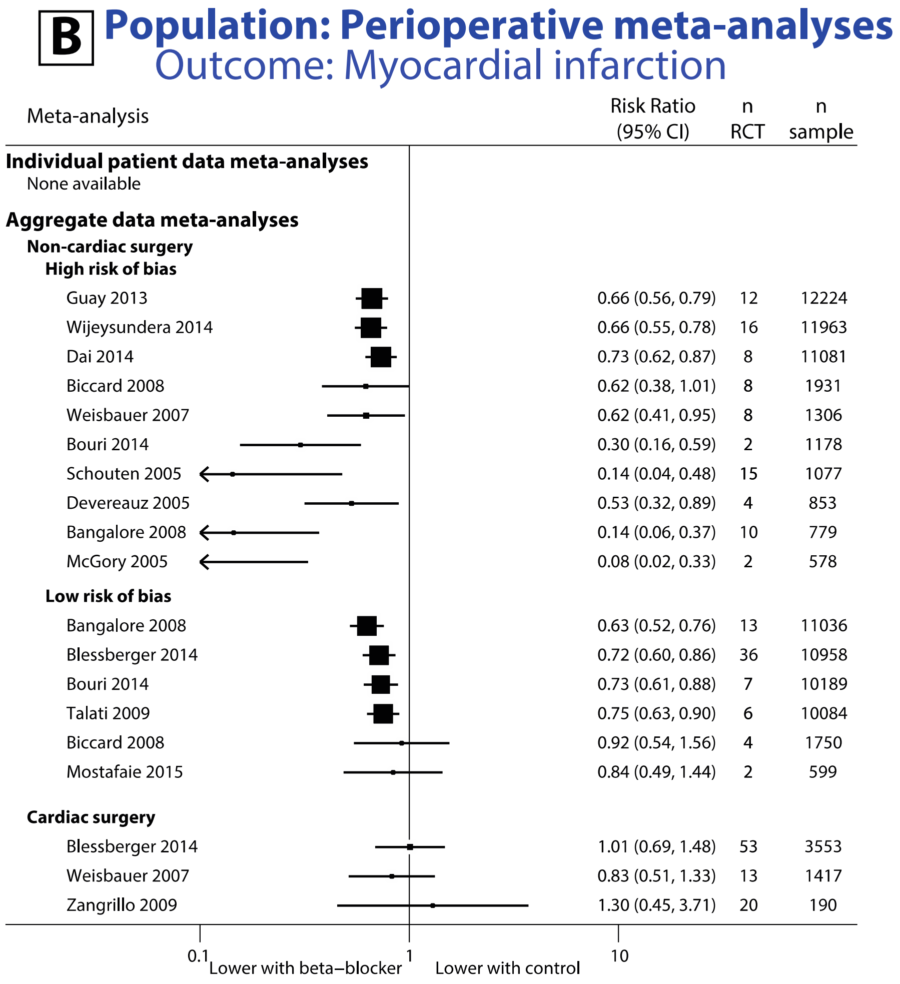

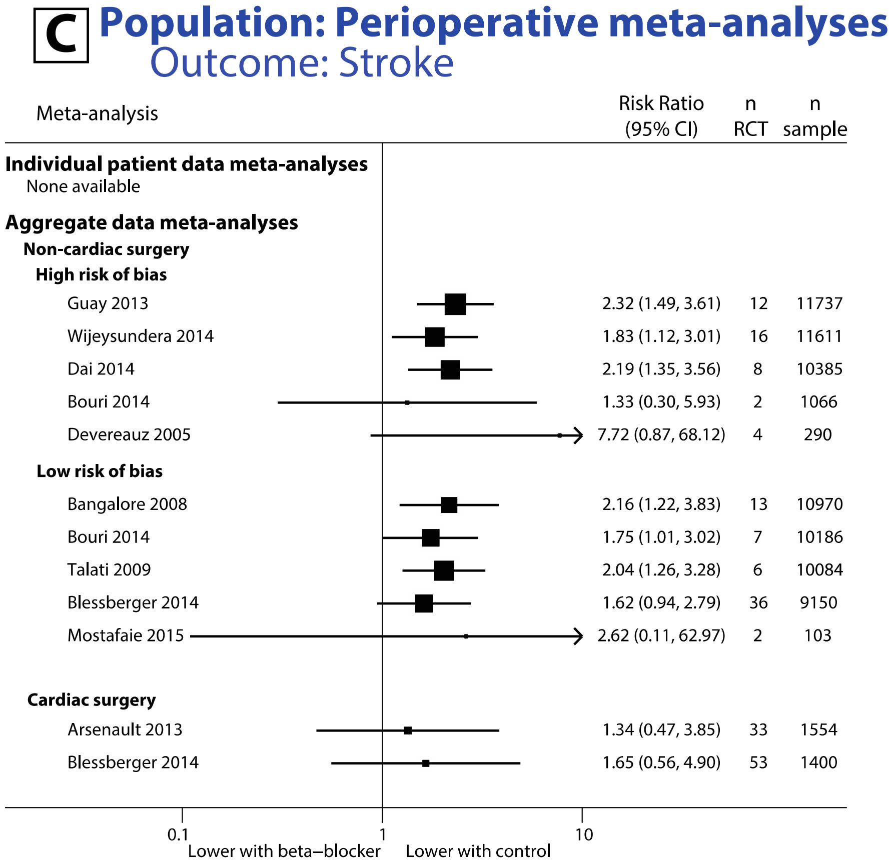

Summary plots of meta-analyses for perioperative risk reduction, including A) all-cause mortality; B) myocardial infarction; and C) stroke; ordered by study quality using AMSTAR index.

Supplement Figure 6: Hypertension meta-analyses

A Population: Hypertension meta-analyses

Outcome: All-cause mortality

Meta-analysis	Participants	Risk Ratio (95% CI)	n RCT	n sample	Follow-up years	AMSTAR
**Individual patient data meta-analyses**						
None available						
**Aggregate data meta-analyses**						
**Beta blocker vs Placebo**						
Bradley 2006	BB used first line	0.96 (0.86, 1.08)	4	23613	5	11/11
Wiysonge 2012	BB first–line, >1yr treatment duration	0.96 (0.86, 1.08)	4	23613	4.85	11/11
Wright 2009	BB first line	1.06 (0.95, 1.19)	5	19313	4.5	11/11
De Lima Luiz 2014	Prior stroke/TIA	0.96 (0.75, 1.23)	2	2193	3	11/11
Psaty 1997	Prior cardiovascular disease	0.99 (0.87, 1.11)	4	18883	1.5	8/11
Khan (<60yr) 2006	<60yr, BB used first line	0.97 (0.82, 1.14)	2	19414	3	6/11
Carlberg 2004	Atenolol used first line	0.98 (0.86, 1.11)	8	6392	4.6	6/11
Wright 1999	BB first line	1.00 (0.87, 1.15)	2	16372	5.03	6/11
Khan (>60yr) 2006	>60yr, BB used first line	0.89 (0.79, 1.01)	5	8019	3	6/11
Kuyper (non-atenolol) 2014	Atenolol used first line	0.97 (0.82, 1.14)	3	19414	n/s	5/11
Kuyper (atenolol) 2014	Atenolol used first line	0.89 (0.79, 1.01)	4	8019	n/s	5/11
Bangalore 2007	Reporting new onset diabetes*	1.04 (0.98, 1.10)	12	n/s	4	5/11
Lindholm 2005	BB used first line in >50%	0.97 (0.88, 1.07)	7	27433	4.90	4/11
Messerli 1998	>60yr, BB/diuretic used first line	1.04 (0.89, 1.21)	2	4199	5.1	4/11
**Beta blocker vs Diuretic**						
Wiysonge 2012	BB first line, >1yr treatment duration	1.04 (0.90, 1.20)	4	18241	4.85	11/11
Bradley 2006	BB used first line	1.04 (0.90, 1.19)	4	17558	5	11/11
Balamuthusamy 2009	Diabetic patients	1.01 (0.92, 1.12)	8	10353	5.4	7/11
Wright 1999	BB first line	1.04 (0.90, 1.20)	2	17952	5.03	6/11
**Beta blocker vs RAS antagonist**						
Wiysonge 2012	BB first line, >1yr treatment duration	1.10 (0.98, 1.24)	4	10828	4.85	11/11
Bradley 2006	BB used first line	1.08 (0.95, 1.22)	4	10828	5	11/11
Xue 2015	Report BP change	1.13 (0.99, 1.29)	2	9193	4.8	11/11
Sciarretta 2011	High CV risk, >65% hypertensive, >200 pts	1.08 (0.96, 1.22)	3	9951	4.3	8/11
Balamuthusamy 2009	Diabetic patients	1.29 (1.09, 1.52)	8	2995	5.4	7/11
**Beta blocker vs Calcium channel blocker**						
Bradley 2006	BB used first line	1.07 (1.00, 1.14)	4	44825	5	11/11
Wiysonge 2012	BB first line, >1yr treatment duration	1.07 (1.00, 1.14)	4	44825	4.85	11/11
Sciarretta 2011	High CV risk, >65% hypertensive, >200 pts	1.11 (1.01, 1.22)	3	19257	4.3	8/11
**Beta blocker vs RAS antagonist, calcium channel blocker or diuretic**						
Bangalore (>60yr) 2008	<60yr without HF, BB used first line	1.03 (0.99, 1.08)	3	87210	3.5	8/11
Bangalore (<60yr) 2008	<60y without HF, BB used first line	0.96 (0.83, 1.11)	6	18312	3.5	8/11
Bangalore 2008	BB used first line*	0.98 (0.94, 1.02)	9	n/s	3.5	7/11
Balamuthusamy 2009	Diabetic patients	1.01 (0.92, 1.12)	8	10353	5.4	7/11
Khan (>60yr) 2006	>60yr, BB used first line	0.98 (0.93, 1.03)	5	n/s	3	6/11
Khan (<60yr) 2006	<60yr, BB used first line	1.06 (0.97, 1.16)	2	n/s	3	6/11
Carlberg 2004	Atenolol used first line	1.13 (1.02, 1.25)	8	17671	4.6	6/11
Kuyper (atenolol) 2014	Non-atenolol BB used first line	0.98 (0.94, 1.03)	4	85960	n/s	5/11
Kuyper (non-atenolol) 2014	Non-atenolol BB used first line	0.96 (0.83, 1.09)	3	21145	n/s	5/11
Lindholm 2005	BB used first line in >50%	0.97 (0.93, 1.01)	7	105951	4.90	4/11
Shinton 1990	Report mortality and stroke outcomes	0.97 (0.84, 1.12)	3	23684	4.43	3/11

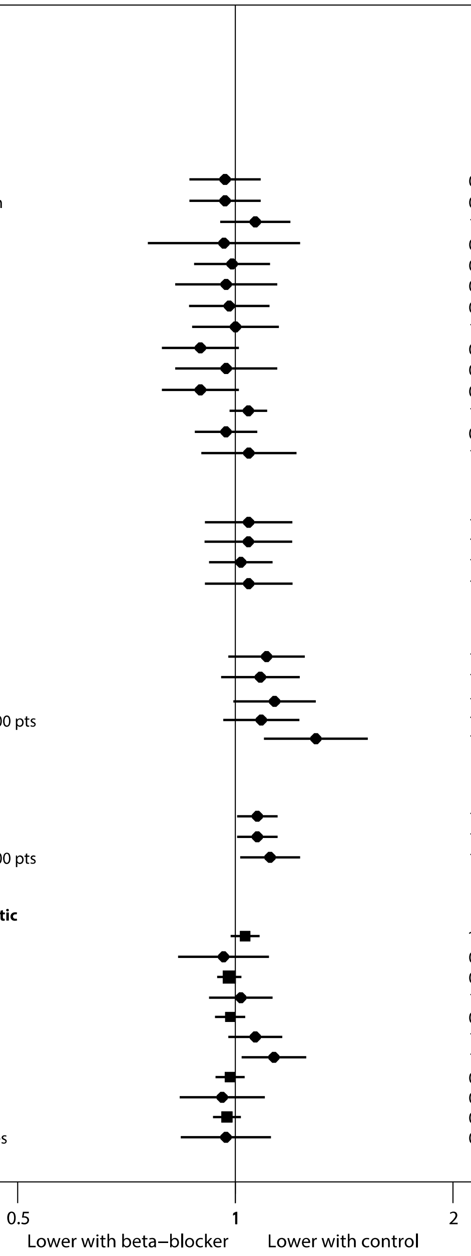

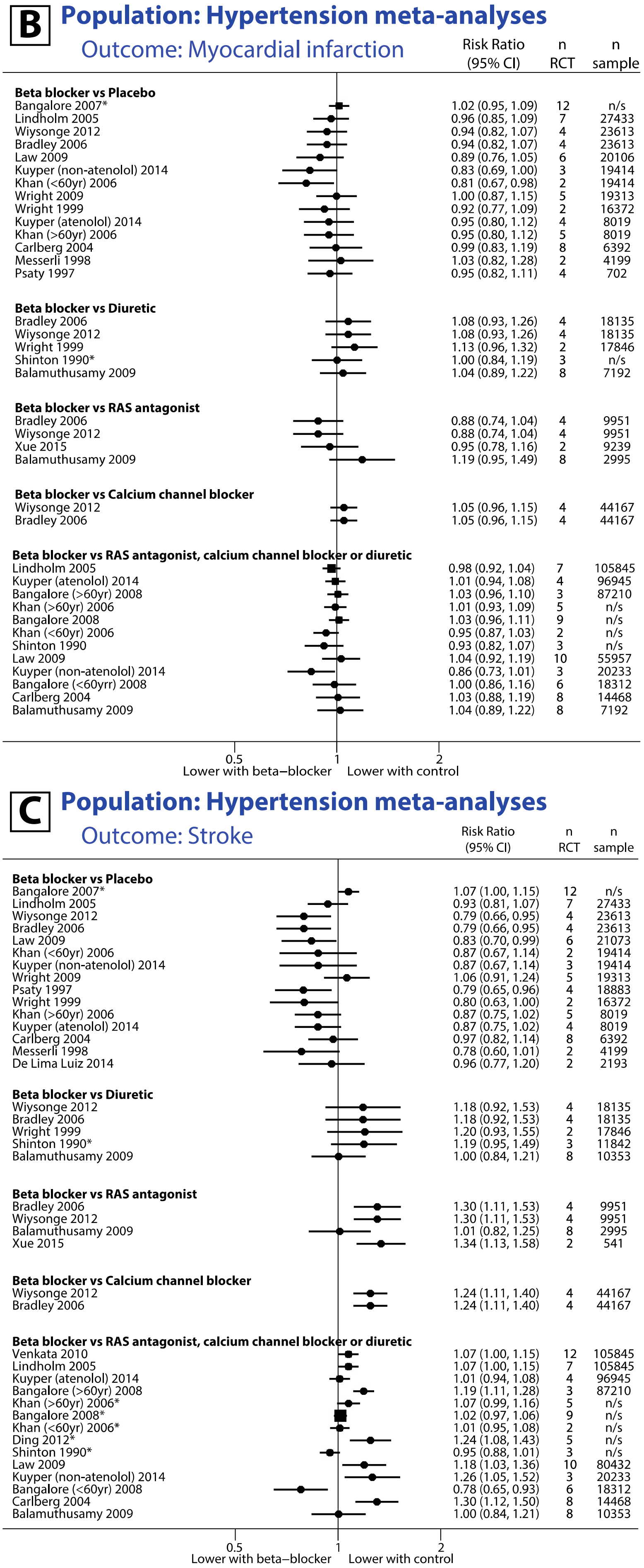

Summary plots of meta-analyses for hypertension reporting A) all-cause mortality; B) myocardial infarction; and C) stroke; ordered by study quality using the AMSTAR index. * adjusted outcome.

Supplemental Figure 7: Hypertension meta-analyses according to beta-blocker type

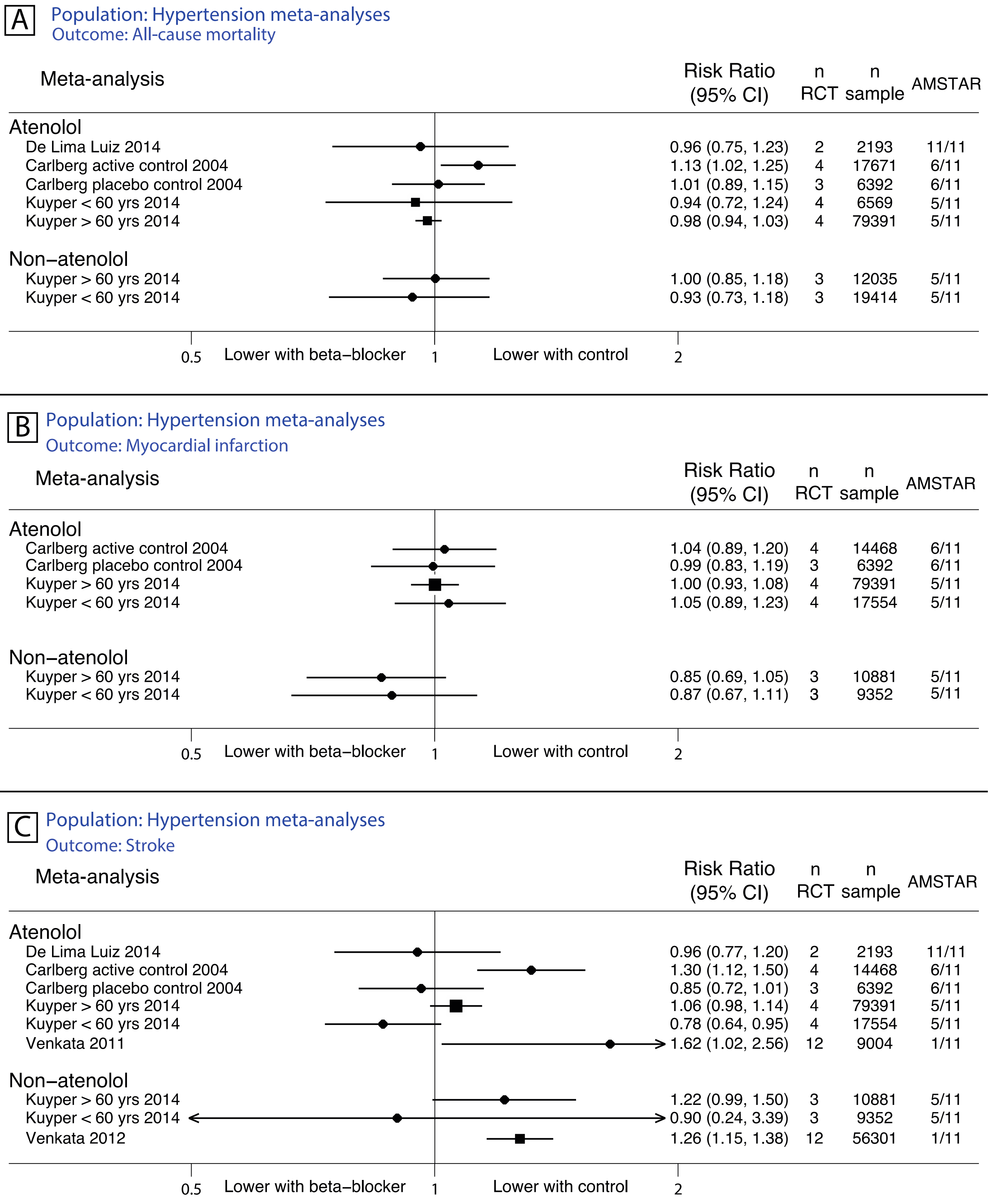

Sensitivity analysis for hypertension according to beta-blocker type (atenolol versus non-atenolol), including A) all-cause mortality; B) myocardial infarction; and C) stroke; ordered by AMSTAR.

Supplemental References

1. Board of Erasmus MC: Follow-up investigation of academic integrity: screening of other publications by Poldermans. Report by second Follow-up Committee published on Erasmus MC website. *Erasmus MC Press Release* 2014.
2. Shea BJ, Hamel C, Wells GA, Bouter LM, Kristjansson E, Grimshaw J, Henry DA, Boers M: AMSTAR is a reliable and valid measurement tool to assess the methodological quality of systematic reviews. *Journal of clinical epidemiology* 2009, 62(10):1013-1020.
3. Whiting P, Savovic J, Higgins JP, Caldwell DM, Reeves BC, Shea B, Davies P, Kleijnen J, Churchill R, group R: ROBIS: A new tool to assess risk of bias in systematic reviews was developed. *Journal of clinical epidemiology* 2016, 69:225-234.
4. Higgins JP, Altman DG, Gotzsche PC, Juni P, Moher D, Oxman AD, Savovic J, Schulz KF, Weeks L, Sterne JA *et al*: The Cochrane Collaboration's tool for assessing risk of bias in randomised trials. *BMJ (Clinical research ed)* 2011, 343:d5928.
5. Zhang J, Yu KF: What's the relative risk? A method of correcting the odds ratio in cohort studies of common outcomes. *JAMA* 1998, 280(19):1690-1691.
6. Al-Reesi A, Al-Zadjali N, Perry J, Fergusson D, Al-Shamsi M, Al-Thagafi M, Stiell I: Do beta-blockers reduce short-term mortality following acute myocardial infarction? A systematic review and meta-analysis. *CJEM* 2008, 10(3):215-223.
7. Bangalore S, Makani H, Radford M, Thakur K, Toklu B, Katz SD, DiNicolantonio JJ, Devereaux PJ, Alexander KP, Wetterslev J *et al*: Clinical outcomes with beta-blockers for myocardial infarction: a meta-analysis of randomized trials. *Am J Med* 2014, 127(10):939-953.
8. Brandler E, Paladino L, Sinert R: Does the early administration of beta-blockers improve the in-hospital mortality rate of patients admitted with acute coronary syndrome? *Acad Emerg Med* 2010, 17(1):1-10.
9. Chatterjee S, Chaudhuri D, Vedanthan R, Fuster V, Ibanez B, Bangalore S, Mukherjee D: Early intravenous beta-blockers in patients with acute coronary syndrome--a meta-analysis of randomized trials. *International journal of cardiology* 2013, 168(2):915-921.
10. Elgendy IY, Elgendy AY, Mahmoud AN, Mansoor H, Mojadidi MK, Bavry AA: Intravenous beta-blockers for patients undergoing primary percutaneous coronary intervention: A meta-analysis of randomized trials. *International journal of cardiology* 2016, 223:891-897.
11. Freemantle N, Cleland J, Young P, Mason J, Harrison J: beta Blockade after myocardial infarction: systematic review and meta regression analysis. *BMJ (Clinical research ed)* 1999, 318(7200):1730-1737.
12. Houghton T, Freemantle N, Cleland JG: Are beta-blockers effective in patients who develop heart failure soon after myocardial infarction? A meta-regression analysis of randomised trials. *Eur J Heart Fail* 2000, 2(3):333-340.
13. Huang HL, Fox KA: The impact of beta-blockers on mortality in stable angina: a meta-analysis. *Scott Med J* 2012, 57(2):69-75.
14. Olsson G, Wikstrand J, Warnold I, Manger Cats V, McBoyle D, Herlitz J, Hjalmarson A, Sonneblick EH: Metoprolol-induced reduction in postinfarction mortality: pooled results from five double-blind randomized trials. *European heart journal* 1992, 13(1):28-32.
15. Paladino L, Sinert R, Brandler E: A review and meta-analysis of studies on the effect and timing of beta-blocker administration in patients with ST-segment elevation myocardial infarction. *Hosp Pract (1995)* 2010, 38(4):63-68.
16. Perez Marco I, Musini Vijaya M, Wright James M: Effect of early treatment with anti-hypertensive drugs on short and long-term mortality in patients with an acute cardiovascular event. In: *Cochrane Database of Systematic Reviews*. John Wiley & Sons, Ltd; 2009.
17. Soriano JB, Hoes AW, Meems L, Grobbee DE: Increased survival with beta-blockers: importance of ancillary properties. *Progress in cardiovascular diseases* 1997, 39(5):445-456.
18. Abdulla J, Kober L, Christensen E, Torp-Pedersen C: Effect of beta-blocker therapy on functional status in patients with heart failure--a meta-analysis. *Eur J Heart Fail* 2006, 8(5):522-531.

19. Al-Gobari M, El Khatib C, Pillon F, Gueyffier F: beta-Blockers for the prevention of sudden cardiac death in heart failure patients: a meta-analysis of randomized controlled trials. *BMC Cardiovasc Disord* 2013, 13:52.
20. Avezum A, Tsuyuki RT, Pogue J, Yusuf S: Beta-blocker therapy for congestive heart failure: a systemic overview and critical appraisal of the published trials. *Can J Cardiol* 1998, 14(8):1045-1053.
21. Badve SV, Roberts MA, Hawley CM, Cass A, Garg AX, Krum H, Tonkin A, Perkovic V: Effects of beta-adrenergic antagonists in patients with chronic kidney disease: a systematic review and meta-analysis. *Journal of the American College of Cardiology* 2011, 58(11):1152-1161.
22. Bavishi C, Chatterjee S, Ather S, Patel D, Messerli FH: Beta-blockers in heart failure with preserved ejection fraction: a meta-analysis. *Heart Fail Rev* 2015, 20(2):193-201.
23. Bell DS, Lukas MA, Holdbrook FK, Fowler MB: The effect of carvedilol on mortality risk in heart failure patients with diabetes: results of a meta-analysis. *Curr Med Res Opin* 2006, 22(2):287-296.
24. Burnett H, Earley A, Voors AA, Senni M, McMurray JJ, Deschaseaux C, Cope S: Thirty Years of Evidence on the Efficacy of Drug Treatments for Chronic Heart Failure With Reduced Ejection Fraction: A Network Meta-Analysis. *Circulation Heart failure* 2017, 10(1).
25. Bonet S, Agusti A, Arnau JM, Vidal X, Diogene E, Galve E, Laporte JR: Beta-adrenergic blocking agents in heart failure: benefits of vasodilating and non-vasodilating agents according to patients' characteristics: a meta-analysis of clinical trials. *Arch Intern Med* 2000, 160(5):621-627.
26. Bouzamondo A, Hulot JS, Sanchez P, Lechat P: Beta-blocker benefit according to severity of heart failure. *Eur J Heart Fail* 2003, 5(3):281-289.
27. Brophy JM, Joseph L, Rouleau JL: Beta-blockers in congestive heart failure. A Bayesian meta-analysis. *Ann Intern Med* 2001, 134(7):550-560.
28. Cleland JGF, Bunting KV, Flather MD, Altman DG, Holmes J, Coats AJS, Manzano L, McMurray JJV, Ruschitzka F, van Veldhuisen DJ *et al*: Beta-blockers for heart failure with reduced, mid-range, and preserved ejection fraction: an individual patient-level analysis of double-blind randomized trials. *European heart journal* 2018, 39(1):26-35.
29. Cleophas TJ, Zwinderman AH: Beta-blockers and heart failure: meta-analysis of mortality trials. *Int J Clin Pharmacol Ther* 2001, 39(9):383-388.
30. Dulin BR, Haas SJ, Abraham WT, Krum H: Do elderly systolic heart failure patients benefit from beta blockers to the same extent as the non-elderly? Meta-analysis of >12,000 patients in large-scale clinical trials. *The American journal of cardiology* 2005, 95(7):896-898.
31. Fauchier L, Pierre B, de Labriolle A, Babuty D: Comparison of the beneficial effect of beta-blockers on mortality in patients with ischaemic or non-ischaemic systolic heart failure: a meta-analysis of randomised controlled trials. *Eur J Heart Fail* 2007, 9(11):1136-1139.
32. Fukuta H, Goto T, Wakami K, Ohte N: The effect of beta-blockers on mortality in heart failure with preserved ejection fraction: A meta-analysis of observational cohort and randomized controlled studies. *International journal of cardiology* 2017, 228:4-10.
33. Haas SJ, Vos T, Gilbert RE, Krum H: Are β-blockers as efficacious in patients with diabetes mellitus as in patients without diabetes mellitus who have chronic heart failure? A meta-analysis of large-scale clinical trials. *American Heart Journal* 2003, 146(5):848-853.
34. Heidenreich PA, Lee TT, Massie BM: Effect of beta-blockade on mortality in patients with heart failure: a meta-analysis of randomized clinical trials. *Journal of the American College of Cardiology* 1997, 30(1):27-34.
35. Kotecha D, Holmes J, Krum H, Altman DG, Manzano L, Cleland JG, Lip GY, Coats AJ, Andersson B, Kirchhof P *et al*: Efficacy of beta blockers in patients with heart failure plus atrial fibrillation: an individual-patient data meta-analysis. *Lancet (London, England)* 2014, 384(9961):2235-2243.
36. Kotecha D, Manzano L, Krum H, Rosano G, Holmes J, Altman DG, Collins PD, Packer M, Wikstrand J, Coats AJ *et al*: Effect of age and sex on efficacy and tolerability of beta blockers in patients with heart failure with reduced ejection fraction: individual patient data meta-analysis. *BMJ (Clinical research ed)* 2016, 353:i1855.

37. Kotecha D, Flather MD, Altman DG, Holmes J, Rosano G, Wikstrand J, Packer M, Coats AJS, Manzano L, Bohm M *et al*: Heart Rate and Rhythm and the Benefit of Beta-Blockers in Patients With Heart Failure. *Journal of the American College of Cardiology* 2017, 69(24):2885-2896.
38. Krum H, Haas SJ, Eichhorn E, Ghali J, Gilbert E, Lechat P, Packer M, Roecker E, Verkenne P, Wedel H *et al*: Prognostic benefit of beta-blockers in patients not receiving ACE-Inhibitors. *European heart journal* 2005, 26(20):2154-2158.
39. Lechat P, Packer M, Chalon S, Cucherat M, Arab T, Boissel JP: Clinical effects of beta-adrenergic blockade in chronic heart failure: a meta-analysis of double-blind, placebo-controlled, randomized trials. *Circulation* 1998, 98(12):1184-1191.
40. Lee S, Spencer A: Beta-blockers to reduce mortality in patients with systolic dysfunction: a meta-analysis. *J Fam Pract* 2001, 50(6):499-504.
41. Liu F, Chen Y, Feng X, Teng Z, Yuan Y, Bin J: Effects of beta-blockers on heart failure with preserved ejection fraction: a meta-analysis. *PLoS One* 2014, 9(3):e90555.
42. Martin N, Manoharan K, Thomas J, Davies C, Lumbers RT: Beta-blockers and inhibitors of the renin-angiotensin aldosterone system for chronic heart failure with preserved ejection fraction. *The Cochrane database of systematic reviews* 2018, 6:CD012721.
43. McAlister FA, Wiebe N, Ezekowitz JA, Leung AA, Armstrong PW: Meta-analysis: beta-blocker dose, heart rate reduction, and death in patients with heart failure. *Ann Intern Med* 2009, 150(11):784-794.
44. Nasr IA, Bouzamondo A, Hulot JS, Dubourg O, Le Heuzey JY, Lechat P: Prevention of atrial fibrillation onset by beta-blocker treatment in heart failure: a meta-analysis. *European heart journal* 2007, 28(4):457-462.
45. O'Connor CM, Fiuzat M, Swedberg K, Caron M, Koch B, Carson PE, Gattis-Stough W, Davis GW, Bristow MR: Influence of global region on outcomes in heart failure beta-blocker trials. *Journal of the American College of Cardiology* 2011, 58(9):915-922.
46. Rienstra M, Damman K, Mulder BA, Van Gelder IC, McMurray JJ, Van Veldhuisen DJ: Beta-blockers and outcome in heart failure and atrial fibrillation: a meta-analysis. *JACC Heart Fail* 2013, 1(1):21-28.
47. Shekelle PG, Rich MW, Morton SC, Atkinson CS, Tu W, Maglione M, Rhodes S, Barrett M, Fonarow GC, Greenberg B *et al*: Efficacy of angiotensin-converting enzyme inhibitors and beta-blockers in the management of left ventricular systolic dysfunction according to race, gender, and diabetic status: a meta-analysis of major clinical trials. *Journal of the American College of Cardiology* 2003, 41(9):1529-1538.
48. Shibata MC, Flather MD, Wang D: Systematic review of the impact of beta blockers on mortality and hospital admissions in heart failure. *Eur J Heart Fail* 2001, 3(3):351-357.
49. van Veldhuisen DJ, McMurray JJ: Pharmacological treatment of heart failure with preserved ejection fraction: a glimpse of light at the end of the tunnel? *Eur J Heart Fail* 2013, 15(1):5-8.
50. Wali RK, Iyengar M, Beck GJ, Chartyan DM, Chonchol M, Lukas MA, Cooper C, Himmelfarb J, Weir MR, Berl T *et al*: Efficacy and safety of carvedilol in treatment of heart failure with chronic kidney disease: a meta-analysis of randomized trials. *Circulation Heart failure* 2011, 4(1):18-26.
51. Whorlow SL, Krum H: Meta-analysis of effect of beta-blocker therapy on mortality in patients with New York Heart Association class IV chronic congestive heart failure. *The American journal of cardiology* 2000, 86(8):886-889.
52. Zaman S, Zaman SS, Scholtes T, Shun-Shin MJ, Plymen CM, Francis DP, Cole GD: The mortality risk of deferring optimal medical therapy in heart failure: a systematic comparison against norms for surgical consent and patient information leaflets. *Eur J Heart Fail* 2017, 19(11):1401-1409.
53. Angeli F, Verdecchia P, Karthikeyan G, Mazzotta G, Gentile G, Reboldi G: ss-Blockers reduce mortality in patients undergoing high-risk non-cardiac surgery. *Am J Cardiovasc Drugs* 2010, 10(4):247-259.
54. Angeli F, Verdecchia P, Karthikeyan G, Mazzotta G, Repaci S, del Pinto M, Gentile G, Cavallini C, Reboldi G: Beta-blockers and risk of all-cause mortality in non-cardiac surgery. *Ther Adv Cardiovasc Dis* 2010, 4(2):109-118.

55. Arsenault KA, Yusuf AM, Crystal E, Healey JS, Morillo CA, Nair GM, Whitlock RP: Interventions for preventing post-operative atrial fibrillation in patients undergoing heart surgery. *The Cochrane database of systematic reviews* 2013(1):CD003611.
56. Badgett RG, Lawrence VA, Cohn SL: Variations in pharmacology of beta-blockers may contribute to heterogeneous results in trials of perioperative beta-blockade. *Anesthesiology* 2010, 113(3):585-592.
57. Bangalore S, Wetterslev J, Pranesh S, Sawhney S, Gluud C, Messerli FH: Perioperative beta blockers in patients having non-cardiac surgery: a meta-analysis. *Lancet (London, England)* 2008, 372(9654):1962-1976.
58. Biccard BM, Sear JW, Foex P: Meta-analysis of the effect of heart rate achieved by perioperative beta-adrenergic blockade on cardiovascular outcomes. *Br J Anaesth* 2008, 100(1):23-28.
59. Blessberger H, Kammler J, Domanovits H, Schlager O, Wildner B, Azar D, Schillinger M, Wiesbauer F, Steinwender C: Perioperative beta-blockers for preventing surgery-related mortality and morbidity. In: *Cochrane Database of Systematic Reviews.* John Wiley & Sons, Ltd; 2014.
60. Bouri S, Shun-Shin MJ, Cole GD, Mayet J, Francis DP: Meta-analysis of secure randomised controlled trials of beta-blockade to prevent perioperative death in non-cardiac surgery. *Heart* 2014, 100(6):456-464.
61. Dai N, Xu D, Zhang J, Wei Y, Li W, Fan B, Xu Y: Different beta-blockers and initiation time in patients undergoing noncardiac surgery: a meta-analysis. *Am J Med Sci* 2014, 347(3):235-244.
62. Devereaux PJ, Beattie WS, Choi PT, Badner NH, Guyatt GH, Villar JC, Cina CS, Leslie K, Jacka MJ, Montori VM *et al*: How strong is the evidence for the use of perioperative beta blockers in non-cardiac surgery? Systematic review and meta-analysis of randomised controlled trials. *BMJ (Clinical research ed)* 2005, 331(7512):313-321.
63. Guay J, Ochroch EA: beta-blocking agents for surgery: influence on mortality and major outcomes. A meta-analysis. *J Cardiothorac Vasc Anesth* 2013, 27(5):834-844.
64. Ji T, Feng C, Sun L, Ye X, Bai Y, Chen Q, Qin Y, Zhu J, Zhao X: Are beta-blockers effective for preventing post-coronary artery bypass grafting atrial fibrillation? Direct and network meta-analyses. *Ir J Med Sci* 2016, 185(2):503-511.
65. Khan MF, Wendel CS, Movahed MR: Prevention of post-coronary artery bypass grafting (CABG) atrial fibrillation: efficacy of prophylactic beta-blockers in the modern era: a meta-analysis of latest randomized controlled trials. *Ann Noninvasive Electrocardiol* 2013, 18(1):58-68.
66. Landoni G, Turi S, Biondi-Zoccai G, Bignami E, Testa V, Belloni I, Cornero G, Zangrillo A: Esmolol reduces perioperative ischemia in noncardiac surgery: a meta-analysis of randomized controlled studies. *J Cardiothorac Vasc Anesth* 2010, 24(2):219-229.
67. McGory ML, Maggard MA, Ko CY: A meta-analysis of perioperative beta blockade: what is the actual risk reduction? *Surgery* 2005, 138(2):171-179.
68. Mostafaie K, Bedenis R, Harrington D: Beta-adrenergic blockers for perioperative cardiac risk reduction in people undergoing vascular surgery. In: *Cochrane Database of Systematic Reviews.* vol. 10.1002/14651858.CD006342.pub2: John Wiley & Sons, Ltd; 2015.
69. Sakamoto A, Hamasaki T, Kitakaze M: Perioperative landiolol administration reduces atrial fibrillation after cardiac surgery: A meta-analysis of randomized controlled trials. *Adv Ther* 2014, 31(4):440-450.
70. Schouten O, Shaw LJ, Boersma E, Bax JJ, Kertai MD, Feringa HH, Biagini E, Kok NF, Urk H, Elhendy A *et al*: A meta-analysis of safety and effectiveness of perioperative beta-blocker use for the prevention of cardiac events in different types of noncardiac surgery. *Coronary artery disease* 2006, 17(2):173-179.
71. Talati R, Reinhart KM, White CM, Phung OJ, Sedrakyan A, Kluger J, Coleman CI: Outcomes of perioperative beta-blockade in patients undergoing noncardiac surgery: a meta-analysis. *Ann Pharmacother* 2009, 43(7):1181-1188.
72. Wang HS, Wang ZW, Yin ZT: Carvedilol for prevention of atrial fibrillation after cardiac surgery: a meta-analysis. *PLoS One* 2014, 9(4):e94005.

73. Wiesbauer F, Schlager O, Domanovits H, Wildner B, Maurer G, Muellner M, Blessberger H, Schillinger M: Perioperative beta-blockers for preventing surgery-related mortality and morbidity: a systematic review and meta-analysis. *Anesth Analg* 2007, 104(1):27-41.
74. Wijeysundera DN, Duncan D, Nkonde-Price C, Virani SS, Washam JB, Fleischmann KE, Fleisher LA, Members AATF: Perioperative beta blockade in noncardiac surgery: a systematic review for the 2014 ACC/AHA guideline on perioperative cardiovascular evaluation and management of patients undergoing noncardiac surgery: a report of the American College of Cardiology/American Heart Association Task Force on Practice Guidelines. *Circulation* 2014, 130(24):2246-2264.
75. Zangrillo A, Turi S, Crescenzi G, Oriani A, Distaso F, Monaco F, Bignami E, Landoni G: Esmolol reduces perioperative ischemia in cardiac surgery: a meta-analysis of randomized controlled studies. *J Cardiothorac Vasc Anesth* 2009, 23(5):625-632.
76. Balamuthusamy S, Molnar J, Adigopula S, Arora R: Comparative analysis of beta-blockers with other antihypertensive agents on cardiovascular outcomes in hypertensive patients with diabetes mellitus: a systematic review and meta-analysis. *American journal of therapeutics* 2009, 16(2):133-142.
77. Bangalore S, Parkar S, Grossman E, Messerli FH: A meta-analysis of 94,492 patients with hypertension treated with beta blockers to determine the risk of new-onset diabetes mellitus. *The American journal of cardiology* 2007, 100(8):1254-1262.
78. Bangalore S, Sawhney S, Messerli FH: Relation of beta-blocker-induced heart rate lowering and cardioprotection in hypertension. *Journal of the American College of Cardiology* 2008, 52(18):1482-1489.
79. Bangalore S, Wild D, Parkar S, Kukin M, Messerli FH: Beta-Blockers for Primary Prevention of Heart Failure in Patients With Hypertension. *Journal of the American College of Cardiology* 2008, 52(13):1062-1072.
80. Bradley HA, Wiysonge CS, Volmink JA, Mayosi BM, Opie LH: How strong is the evidence for use of beta-blockers as first-line therapy for hypertension? Systematic review and meta-analysis. *J Hypertens* 2006, 24(11):2131-2141.
81. Carlberg B, Samuelsson O, Lindholm LH: Atenolol in hypertension: is it a wise choice? *Lancet (London, England)* 2004, 364(9446):1684-1689.
82. Cruickshank JM: The Role of Beta-Blockers in the Treatment of Hypertension. *Advances in experimental medicine and biology* 2017, 956:149-166.
83. De Lima Luiz G, Saconato H, Atallah Álvaro N, da Silva Edina MK: Beta-blockers for preventing stroke recurrence. In: *Cochrane Database of Systematic Reviews.* vol. 10.1002/14651858.CD007890.pub3: John Wiley & Sons, Ltd; 2014.
84. Ding FH, Li Y, Li LH, Wang JG: Impact of heart rate on central hemodynamics and stroke: a meta-analysis of beta-blocker trials. *Am J Hypertens* 2013, 26(1):118-125.
85. Jeffers BW, Robbins J, Bhambri R: Efficacy of Calcium Channel Blockers Versus Other Classes of Antihypertensive Medication in the Treatment of Hypertensive Patients With Previous Stroke and/or Coronary Artery Disease: A Systematic Review and Meta-Analysis. *American journal of therapeutics* 2017, 24(1):e68-e80.
86. Khan N, McAlister FA: Re-examining the efficacy of beta-blockers for the treatment of hypertension: a meta-analysis. *CMAJ : Canadian Medical Association journal = journal de l'Association medicale canadienne* 2006, 174(12):1737-1742.
87. Kuyper LM, Khan NA: Atenolol vs nonatenolol beta-blockers for the treatment of hypertension: a meta-analysis. *Can J Cardiol* 2014, 30(5 Suppl):S47-53.
88. Law MR, Morris JK, Wald NJ: Use of blood pressure lowering drugs in the prevention of cardiovascular disease: meta-analysis of 147 randomised trials in the context of expectations from prospective epidemiological studies. *BMJ (Clinical research ed)* 2009, 338:b1665.
89. Lindholm LH, Carlberg B, Samuelsson O: Should beta blockers remain first choice in the treatment of primary hypertension? A meta-analysis. *Lancet (London, England)* 2005, 366(9496):1545-1553.
90. Messerli FH, Grossman E, Goldbourt U: Are beta-blockers efficacious as first-line therapy for hypertension in the elderly? A systematic review. *JAMA* 1998, 279(23):1903-1907.

91. Palla M, Ando T, Androulakis E, Telila T, Briasoulis A: Renin-Angiotensin System Inhibitors vs Other Antihypertensives in Hypertensive Blacks: A Meta-Analysis. *Journal of clinical hypertension (Greenwich, Conn)* 2017, 19(4):344-350.
92. Psaty BM, Smith NL, Siscovick DS, Koepsell TD, Weiss NS, Heckbert SR, Lemaitre RN, Wagner EH, Furberg CD: Health outcomes associated with antihypertensive therapies used as first-line agents. A systematic review and meta-analysis. *JAMA* 1997, 277(9):739-745.
93. Remonti LR, Dias S, Leitao CB, Kramer CK, Klassman LP, Welton NJ, Ades AE, Gross JL: Classes of antihypertensive agents and mortality in hypertensive patients with type 2 diabetes-Network meta-analysis of randomized trials. *Journal of diabetes and its complications* 2016, 30(6):1192-1200.
94. Sciarretta S, Palano F, Tocci G, Baldini R, Volpe M: Antihypertensive treatment and development of heart failure in hypertension: a Bayesian network meta-analysis of studies in patients with hypertension and high cardiovascular risk. *Arch Intern Med* 2011, 171(5):384-394.
95. Shinton RA, Beevers DG: A meta-analysis of mortality and coronary prevention in hypertensive patients treated with beta-receptor blockers. *J Hum Hypertens* 1990, 4 Suppl 2:31-34.
96. Ram CV: Beta-blockers in hypertension. *The American journal of cardiology* 2010, 106(12):1819-1825.
97. Wang WT, You LK, Chiang CE, Sung SH, Chuang SY, Cheng HM, Chen CH: Comparative Effectiveness of Blood Pressure-lowering Drugs in Patients who have Already Suffered From Stroke: Traditional and Bayesian Network Meta-analysis of Randomized Trials. *Medicine* 2016, 95(15):e3302.
98. Wiysonge CS, Bradley HA, Volmink J, Mayosi BM, Mbewu A, Opie LH: Beta-blockers for hypertension. *The Cochrane database of systematic reviews* 2012, 11:CD002003.
99. Wiysonge CS, Bradley HA, Volmink J, Mayosi BM, Opie LH: Beta-blockers for hypertension. *The Cochrane database of systematic reviews* 2017, 1:CD002003.
100. Wright JM, Lee CH, Chambers GK: Systematic review of antihypertensive therapies: does the evidence assist in choosing a first-line drug? *CMAJ : Canadian Medical Association journal = journal de l'Association medicale canadienne* 1999, 161(1):25-32.
101. Wright JM: Choosing a first-line drug in the management of elevated blood pressure: what is the evidence? 2: Beta-blockers. *CMAJ : Canadian Medical Association journal = journal de l'Association medicale canadienne* 2000, 163(2):188-192.
102. Wright James M, Musini Vijaya M: First-line drugs for hypertension. In: *Cochrane Database of Systematic Reviews.* John Wiley & Sons, Ltd; 2009.
103. Xue H, Lu Z, Tang Wen L, Pang Lu W, Wang Gan M, Wong Gavin WK, Wright James M: First-line drugs inhibiting the renin angiotensin system versus other first-line antihypertensive drug classes for hypertension. In: *Cochrane Database of Systematic Reviews.* John Wiley & Sons, Ltd; 2015.
104. Heidenreich PA, McDonald KM, Hastie T, Fadel B, Hagan V, Lee BK, Hlatky MA: Meta-analysis of trials comparing beta-blockers, calcium antagonists, and nitrates for stable angina. *JAMA* 1999, 281(20):1927-1936.
105. Howes LG, Lykos D, Rennie GC: Effects of antihypertensive drugs on coronary artery disease risk: a meta-analysis. *Clin Exp Pharmacol Physiol* 1996, 23(6-7):555-558.
106. Huang BT, Huang FY, Zuo ZL, Liao YB, Heng Y, Wang PJ, Gui YY, Xia TL, Xin ZM, Liu W *et al*: Meta-Analysis of Relation Between Oral beta-Blocker Therapy and Outcomes in Patients With Acute Myocardial Infarction Who Underwent Percutaneous Coronary Intervention. *The American journal of cardiology* 2015, 115(11):1529-1538.
107. Jia Y, Leung SW: Comparative Efficacy of Tongxinluo Capsule and Beta-Blockers in Treating Angina Pectoris: Meta-Analysis of Randomized Controlled Trials. *J Altern Complement Med* 2015, 21(11):686-699.
108. Misumida N, Harjai K, Kernis S, Kanei Y: Does Oral Beta-Blocker Therapy Improve Long-Term Survival in ST-Segment Elevation Myocardial Infarction With Preserved Systolic Function? A Meta-Analysis. *J Cardiovasc Pharmacol Ther* 2016, 21(3):280-285.
109. Shu de F, Dong BR, Lin XF, Wu TX, Liu GJ: Long-term beta blockers for stable angina: systematic review and meta-analysis. *Eur J Prev Cardiol* 2012, 19(3):330-341.

110. Briasoulis A, Palla M, Afonso L: Meta-analysis of the effects of carvedilol versus metoprolol on all-cause mortality and hospitalizations in patients with heart failure. *The American journal of cardiology* 2015, 115(8):1111-1115.
111. Chatterjee S, Biondi-Zoccai G, Abbate A, D'Ascenzo F, Castagno D, Van Tassell B, Mukherjee D, Lichstein E: Benefits of beta blockers in patients with heart failure and reduced ejection fraction: network meta-analysis. *BMJ (Clinical research ed)* 2013, 346:f55.
112. DiNicolantonio JJ, Lavie CJ, Fares H, Menezes AR, O'Keefe JH: Meta-analysis of carvedilol versus beta 1 selective beta-blockers (atenolol, bisoprolol, metoprolol, and nebivolol). [Review]. 2013.
113. Dobre D, Haaijer-Ruskamp FM, Voors AA, van Veldhuisen DJ: beta-Adrenoceptor antagonists in elderly patients with heart failure: a critical review of their efficacy and tolerability. *Drugs Aging* 2007, 24(12):1031-1044.
114. Leizorovicz A, Lechat P, Cucherat M, Bugnard F: Bisoprolol for the treatment of chronic heart failure: a meta-analysis on individual data of two placebo-controlled studies--CIBIS and CIBIS II. Cardiac Insufficiency Bisoprolol Study. *Am Heart J* 2002, 143(2):301-307.
115. Packer M, Antonopoulos GV, Berlin JA, Chittams J, Konstam MA, Udelson JE: Comparative effects of carvedilol and metoprolol on left ventricular ejection fraction in heart failure: results of a meta-analysis. *Am Heart J* 2001, 141(6):899-907.
116. Prins KW, Neill JM, Tyler JO, Eckman PM, Duval S: Effects of Beta-Blocker Withdrawal in Acute Decompensated Heart Failure: A Systematic Review and Meta-Analysis.[Erratum appears in JACC Heart Fail. 2015 Oct;3(10):847]. 2015.
117. Zarembski DG, Nolan PE, Jr., Slack MK, Lui CY: Meta-analysis of the use of low-dose beta-adrenergic blocking therapy in idiopathic or ischemic dilated cardiomyopathy. *The American journal of cardiology* 1996, 77(14):1247-1250.
118. Crystal E, Connolly SJ, Sleik K, Ginger TJ, Yusuf S: Interventions on prevention of postoperative atrial fibrillation in patients undergoing heart surgery: a meta-analysis. *Circulation* 2002, 106(1):75-80.
119. DiNicolantonio JJ, Beavers CJ, Menezes AR, Lavie CJ, O'Keefe JH, Meier P, Vorobcsuk A, Aradi D, Komocsi A, Chatterjee S *et al*: Meta-analysis comparing carvedilol versus metoprolol for the prevention of postoperative atrial fibrillation following coronary artery bypass grafting. *The American journal of cardiology* 2014, 113(3):565-569.
120. Kaw R, Hernandez AV, Masood I, Gillinov AM, Saliba W, Blackstone EH: Short- and long-term mortality associated with new-onset atrial fibrillation after coronary artery bypass grafting: a systematic review and meta-analysis. *J Thorac Cardiovasc Surg* 2011, 141(5):1305-1312.
121. Ollila A, Vikatmaa L, Sund R, Pettila V, Wilkman E: Efficacy and safety of intravenous esmolol for cardiac protection in non-cardiac surgery. A systematic review and meta-analysis. *Annals of medicine* 2019, 51(1):17-27.
122. Yu SK, Tait G, Karkouti K, Wijeysundera D, McCluskey S, Beattie WS: The safety of perioperative esmolol: a systematic review and meta-analysis of randomized controlled trials. *Anesth Analg* 2011, 112(2):267-281.
123. Aursnes I, Tvete IF, Gasemyr J, Natvig B: Clinical efficacies of antihypertensive drugs. *Scand Cardiovasc J* 2003, 37(2):72-79.
124. Baguet JP, Robitail S, Boyer L, Debensason D, Auquier P: A meta-analytical approach to the efficacy of antihypertensive drugs in reducing blood pressure. *Am J Cardiovasc Drugs* 2005, 5(2):131-140.
125. Baguet JP, Legallicier B, Auquier P, Robitail S: Updated meta-analytical approach to the efficacy of antihypertensive drugs in reducing blood pressure. *Clin Drug Investig* 2007, 27(11):735-753.
126. Dahlof B, Devereux RB, Kjeldsen SE, Lyle PA, Zhang Z, Edelman JM: Atenolol as a comparator in outcome trials in hypertension: a correct choice in the past, but not for the future? *Blood Press* 2007, 16(1):6-12.
127. Germino FW, Lin Y, Pejovic V, Bowen L: Efficacy and tolerability of nebivolol: does age matter? A retrospective analysis of three randomized, placebo-controlled trials in stage I-II hypertension. *Ther Adv Cardiovasc Dis* 2012, 6(5):185-199.

128. Magee LA, Elran E, Bull SB, Logan A, Koren G: Risks and benefits of beta-receptor blockers for pregnancy hypertension: overview of the randomized trials. *Eur J Obstet Gynecol Reprod Biol* 2000, 88(1):15-26.
129. Mulrow C, Lau J, Cornell J, Brand M: Pharmacotherapy for hypertension in the elderly. *The Cochrane database of systematic reviews* 2000(2):CD000028.
130. Psaty BM, Lumley T, Furberg CD, Schellenbaum G, Pahor M, Alderman MH, Weiss NS: Health outcomes associated with various antihypertensive therapies used as first-line agents: a network meta-analysis. *JAMA* 2003, 289(19):2534-2544.
131. Turnbull F, Blood Pressure Lowering Treatment Trialists C: Effects of different blood-pressure-lowering regimens on major cardiovascular events: results of prospectively-designed overviews of randomised trials. *Lancet (London, England)* 2003, 362(9395):1527-1535.
132. Turnbull F, Neal B, Algert C, Chalmers J, Chapman N, Cutler J, Woodward M, MacMahon S, Blood Pressure Lowering Treatment Trialists C: Effects of different blood pressure-lowering regimens on major cardiovascular events in individuals with and without diabetes mellitus: results of prospectively designed overviews of randomized trials. *Arch Intern Med* 2005, 165(12):1410-1419.
133. Schouten O, Shaw LJ, Boersma E, Bax JJ, Kertai MD, Feringa HH, Biagini E, Kok NF, Urk H, Elhendy A *et al*: A meta-analysis of safety and effectiveness of perioperative beta-blocker use for the prevention of cardiac events in different types of noncardiac surgery. *Coron Artery Dis* 2006, 17(2):173-9.
134. Gong Y, Wang Z, Beitelshees AL, McDonough CW, Langaee TY, Hall K, Schmidt SO, Curry RW, Jr., Gums JG, Bailey KR *et al*: Pharmacogenomic Genome-Wide Meta-Analysis of Blood Pressure Response to beta-Blockers in Hypertensive African Americans. *Hypertension (Dallas, Tex : 1979)* 2016, 67(3):556-563.
